# ENACT: End-to-End Analysis of Visium High Definition (HD) Data

**DOI:** 10.1093/bioinformatics/btaf094

**Published:** 2025-03-07

**Authors:** Mena Kamel, Yiwen Song, Ana Solbas, Sergio Villordo, Amrut Sarangi, Pavel Senin, Mathew Sunaal, Luis Cano Ayestas, Clement Levin, Seqian Wang, Marion Classe, Ziv Bar-Joseph, Albert Pla Planas

**Affiliations:** Digital R&D, Sanofi, Toronto, ON M5V 1V6, Canada; Digital R&D, Sanofi, Toronto, ON M5V 1V6, Canada; Digital R&D, Sanofi, Barcelona 08016, Spain; Digital R&D, Sanofi, Barcelona 08016, Spain; Digital R&D, Sanofi, Toronto, ON M5V 1V6, Canada; Digital R&D, Sanofi, Toronto, ON M5V 1V6, Canada; Digital R&D, Sanofi, Toronto, ON M5V 1V6, Canada; Precision Medicine & Computational Biology, Sanofi, Paris 94400, France; Precision Medicine & Computational Biology, Sanofi, Paris 94400, France; Digital R&D, Sanofi, Toronto, ON M5V 1V6, Canada; Precision Medicine & Computational Biology, Sanofi, Paris 94400, France; Digital R&D, Sanofi, Cambridge, MA 02141, United States; Digital R&D, Sanofi, Barcelona 08016, Spain

## Abstract

**Motivation:**

Spatial transcriptomics (ST) enables the study of gene expression within its spatial context in histopathology samples. To date, a limiting factor has been the resolution of sequencing based ST products. The introduction of the Visium High Definition (HD) technology opens the door to cell resolution ST studies. However, challenges remain in the ability to accurately map transcripts to cells and in assigning cell types based on the transcript data.

**Results:**

We developed ENACT, a self-contained pipeline that integrates advanced cell segmentation with Visium HD transcriptomics data to infer cell types across whole tissue sections. Our pipeline incorporates novel bin-to-cell assignment methods, enhancing the accuracy of single-cell transcript estimates. Validated on diverse synthetic and real datasets, our approach is both scalable to samples with hundreds of thousands of cells and effective, offering a robust solution for spatially resolved transcriptomics analysis.

**Availability and implementation:**

ENACT source code is available at https://github.com/Sanofi-Public/enact-pipeline. Experimental data are available at https://zenodo.org/records/14748859.

## 1 Introduction

Spatial transcriptomics (ST) is a promising new technology that enables researchers to explore the spatial distribution of cells within tissues. ST technologies can be divided into two categories; sequencing-based (e.g. Visium and GeoMX) and image-based (e.g. Xenium, MERFISH, and CosMX). Sequencing-based technologies provide several advantages including the ability to comprehensively map the entire transcriptome, identification of splice variants, and computation of RNA-velocity (Chen *et al.* 2023). The data obtained from sequencing-based methods is collected from spots placed in a grid-like manner. These spots typically have a multi-cell resolution, preventing the study of the tissue at a single-cell resolution.

Recently, 10× Genomics released Visium high definition (HD) (Nagendran *et al.* 2023), a new sequencing-based ST platform that collects transcript counts at a sub-cellular resolution. Specifically, spots (also referred to as bins) in Visium HD are 2 × 2 µm. To enable cell-based analysis, Visium HD allows the user to aggregate bins resulting in an 8 × 8 µm dimension for each spot, which roughly covers the size of a cell. However, accurately getting single-cell transcript estimates from the aggregated 8 × 8 µm is not trivial. First, some cells are smaller than the aggregated spot size which leads to contamination. Even more problematic, these aggregated spots rarely completely overlap a single cell. In many cases, each such aggregated spot overlaps 2 or more cells and vice versa, each cell overlaps more than 1 spot (often more than 2 given the 3D placement) especially in cases where cells are smaller than 8 µm in diameter, or where cells overlap or are tightly spaced.

To address this problem, instead of using the 8 × 8 µm bins for downstream analysis, previous work such as Bin2cell (Krzysztof Polanski *et al.* 2024) proposes a method that combines morphology and gene expression information to obtain accurate single-cell transcript counts. Bin2cell segments nuclei using a two-stage process that combines imaging and gene expression information, leveraging Stardist (Schmidt *et al.* 2018). These outlines are then expanded, and the 2 × 2 µm bins with centroids within each cell outline are aggregated and normalized to obtain single-cell transcript counts.

While such integrated imaging plus sequencing method obtains good results in some cases, its accuracy can be reduced in scenarios where cells are tightly packed. In these situations, many bins overlap multiple cells, necessitating a strategy to appropriately assign transcript contributions from these bins to the overlapping cells. Bin2cell assigns overlapping bins exclusively to a single cell based on gene expression proximity in PCA space. Other approaches, such as dividing the contribution of overlapping bins among the cells they intersect, have not been explored to date.

To address these issues, we developed ENACT, a comprehensive pipeline for processing Visium HD results. Our pipeline integrates state-of-the-art deep learning-based cell segmentation models, including Stardist, to predict cell boundaries. Using these predicted boundaries, several bin-to-cell assignment strategies that redistribute the transcripts in the overlapping bins are implemented to obtain accurate single-cell transcript counts. Cell types are subsequently inferred using either CellAssign (Zhang *et al.* 2019a), a probabilistic method; CellTypist (Domínguez Conde *et al.* 2022), a logistic regression model; or Sargent (Nouri *et al.* 2023), a scoring-based approach. With the predicted cell types and their locations, downstream analysis can be conducted on the output AnnData objects using Squidpy (Palla *et al.* 2022).

## 2 Materials and methods

### 2.1 Cell segmentation

**Figure 1.**
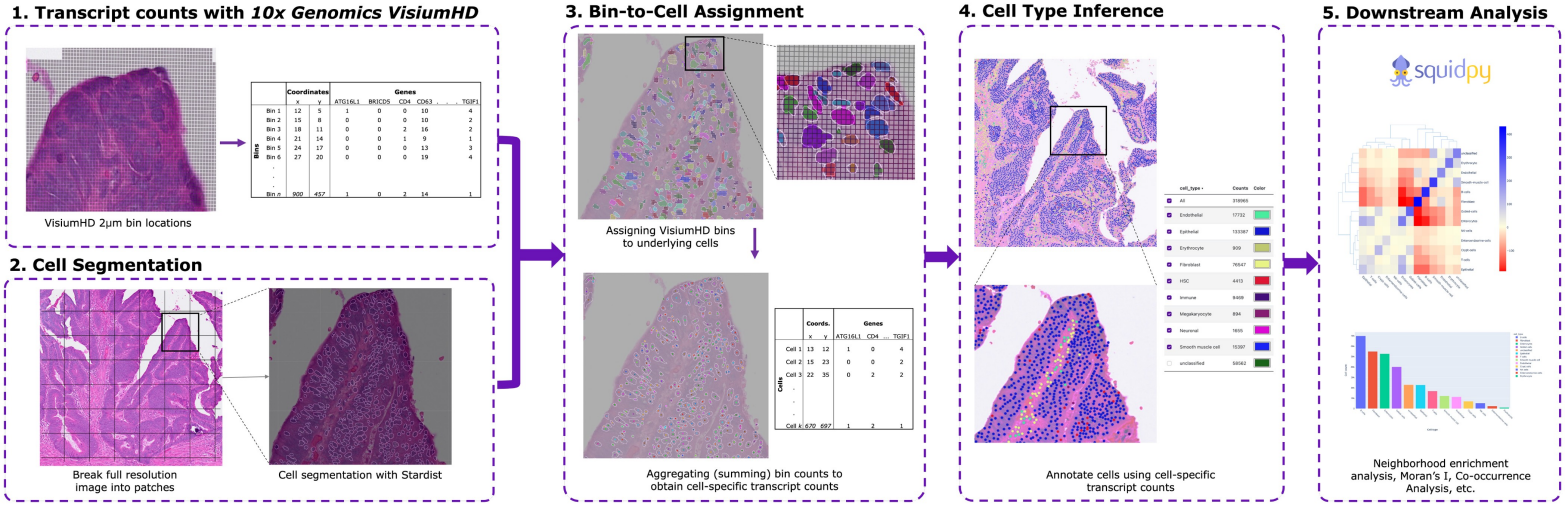
Processing pipeline. (1) Bin-by-gene matrix from 10× Genomics Visium HD provides mRNA transcript counts for each 2 × 2 µm bin. (2) Segmenting the full resolution tissue image using Startdist. (3) Visium HD bins that overlap with a cell are aggregated via a summing operation. This leads to a 2D cell-by-gene matrix. (4) Sargent, CellAssign, or CellTypist are used to translate the cell-wise transcript counts to a respective cell label. (5) Cell labels and their spatial locations within the tissue are wrapped in AnnData objects, which is compatible with SquidPy. This enables the application of various spatial statistical analyses, including neighborhood enrichment, Moran’s I, and co-occurrence analyses. Spatial distribution of the cells can be visualized by Tissuumaps (Pielawski *et al.* 2023) digital pathology viewer.

Figure 1 presents the key steps involved in the proposed processing and analysis pipeline. Cells in the full resolution tissue image are first segmented using Stardist, one of the most widely used UNet-based cell segmentation methods that has been shown to accurately segment images even when cells are tightly packed (Schmidt *et al.* 2018).

### 2.2 Bin-to-cell assignment

To obtain the total transcript counts for each cell in the tissue, the 2 × 2 µm Visium HD bins and the predicted cell outlines are represented as Shapely (Gillies *et al.* 2007) polygons. This enables efficient geometric operations such as computing intersection area and spatial relationships. A spatial join operation is run to identify the spatial relationship between the Visium HD bins and the cell outlines that they intersect. Visium HD bins that do not geometrically intersect with any cell outlines are removed from further analysis. To address bins overlapping multiple cells, we include in ENACT four different transcript imputation methods: a naive method, and three weight-based strategies. Supplementary Section S1 provides figures and details on the implementation of all four bin-to-cell assignment strategies. This produces a 2D cell-by-gene matrix, similar to a single-cell RNA sequencing (scRNA-Seq), mapped to each cell’s location on the image.

### 2.3 Cell type annotation

Several methods are available for cell type annotation using the obtained single-cell expression profiles. We use one of Sargent (SignAtuRe-GEne cell aNnoTation) (Nouri *et al.* 2023), CellAssign (Zhang *et al.* 2019a), or CellTypist (Domínguez Conde *et al.* 2022) to annotate cell types. Sargent is a score-based, single-cell inference algorithm that identifies the cell types of origin based on cell-type specific gene markers. This fits very well for Visium HD data since transcript counts are often low compared to traditional scRNA-Seq experiments, making score-based methods more fitting. CellAssign infers the cell type by computing a probabilistic assignment relative to a cell-type specific gene markers. Lastly, CellTypist is a machine learning-based tool that utilizes classifiers pre-trained on an extensive reference dataset of annotated cells to rapidly assign cell types. CellTypist does not require users to provide gene markers, as they are already defined in the pre-trained models. For Sargent and CellAssign, markers can be obtained from a variety of sources including open-source databases such as PanglaoDB (Franzén *et al.* 2019) or CellMarker (Zhang *et al.* 2019b).

### 2.4 Downstream analysis

The cell labels and their respective locations in the tissue are wrapped in AnnData objects for easy import into open source libraries such as SquidPy. This can be used to run several spatial statistical tests such as neighborhood enrichment analysis, Moran’s I analysis, co-occurrence analysis, etc. (see ENACT’s documentation for examples).

## 3 Evaluation datasets

To evaluate the accuracy of transcript assignment, two synthetic Visium HD-like datasets are constructed from Xenium (Janesick *et al.* 2023) and sequential fluorescence *in situ* hybridization (seqFISH+) (Eng and Cai 2019). In addition, the end-to-end pipeline is evaluated against five public samples from different tissues (human lung, colon, tonsil and breast, and mouse intestine). Expert annotations provided by a pathologist and an immunologist, consisting of anatomical landmarks (labeled tissue regions) and cell type labels, are obtained for all samples. Details on the synthetic dataset generation and expert annotations are provided in Supplementary Section S2.

## 4 Results

### 4.1 Evaluating bin-to-cell assignment methods

While all four possible bin-to-cell assignment methods are available for users, we first evaluate them on two synthetic datasets to provide guidance on their performance. Supplementary Figs S7–S12 provide details about the tissue density and transcript sparsity of the cells that make up the synthetic datasets.

Results for the Xenium-based synthetic dataset with whole cell boundaries are shown in Supplementary Table ST3 and Fig. S18. The ‘Weight-By-Area’ method achieves the highest F1 score among the four approaches when evaluating the entire cell boundary. In contrast, the naive method achieves the highest precision as it only considers the unique bins, omitting all the bins shared with multiple cells. However, this approach results in significant information loss, as ∼25% of the bins overlap with more than one cell, leading to a low recall (Supplementary Table ST3).

When focusing solely on cell nuclei, we observe a smaller difference in performance between the ‘Naive’ and weighted methods (Supplementary Fig. S19). This is likely due to the smaller number of bins overlapping with multiple nuclei, limiting the benefits of the weighted methods over the ‘Naive’ method. Additionally, all methods show lower precision on cell nuclei compared to whole cells, which can be attributed to a higher number of non-overlapping bins located at the boundaries of the nuclei, where some transcripts belong to the cell body rather than to the nucleus.

Similarly, for the seqFISH+ synthetic dataset, all four methods demonstrate high precision (close to 1) and recall (close to 0.99, Supplementary Fig. S20). This performance can likely be attributed to the relatively large size and sparseness of the NIH-3T3 cells, which generally intersect with 200 to 700 bins. Additionally, only about 5% of bins overlapped more than one cell for this dataset. In such cases, the ‘Naive’ method, may offer the best compromise between accuracy and compute time. Supplementary Table ST4 and Fig. S22 present the run times for the different bin-to-cell assignment methods explored.

### 4.2 Evaluating cell-type annotation

#### 4.2.1 Evaluating cell-types in pathologist annotations

To evaluate the impact of different bin-to-cell assignment methods on cell type annotation, we compare the annotations generated by our pipeline, employing the four distinct bin-to-cell assignment strategies, against expert-labeled classifications for 20 991 cells. Supplementary Table ST7 presents the gene markers used by Sargent and CellAssign to annotate the cell types. This list is curated by a combination of expert provided gene markers and PanglaoDB where the top 10 genes by sensitivity and specificity in humans are selected. To align the more granular cell labels from CellAssign, CellTypist, and Sargent with the broader, pathologist-provided cell labels (Epithelial, Stromal, and Immune cells), the cell types are relabeled according to the mapping defined in Supplementary Table ST5.

Supplementary Table ST6 and Fig. S23 summarize the experiments we perform to test the four bin-to-cell assignment methods with the three cell-type annotation algorithms. The performance of the pipeline is assessed as a multi-class classifier, using accuracy, precision, recall, and F1-score (the harmonic mean of precision and recall) as evaluation metrics for the predicted cell types. Supplementary Table ST6 shows that the weighted methods consistently outperform the ‘Naive’ approach across all three cell annotation methods, yielding higher recall, precision, and F1-score values suggesting that a more accurate bin-to-cell assignment method can improve cell annotation. Among the several cell annotation methods, Sargent exhibits the highest performance for annotating cells, followed by CellTypist, with CellAssign ranking last. Sargent achieves the best results when paired with the ‘Weighted-by-Area’ approach, yielding an accuracy of 0.708 and a weighted F1-score of 0.758.

Supplementary Fig. S24 presents the confusion matrices for all twelve experiments, highlighting distinct patterns in cell misclassification across methods. CellAssign fails to identify all immune cells, misclassifying them as epithelial cells. Similarly, CellTypist confuses a significant portion of immune cells being incorrectly labeled as epithelial. In contrast, Sargent shows the best performance, significantly reducing misclassifications and accurately differentiating immune cells from epithelial cells. Errors in the predicted cell types may stem from the cell-specific gene markers used, as these directly influence classification outcomes. Additionally, the manual annotation process, being inherently labor-intensive, may result in mislabeled cells, contributing to the observed inaccuracies.

#### 4.2.2 Evaluating cell-types in anatomical landmarks

To assess the high-level performance of the proposed pipeline, we analyze the assigned cell types within each anatomical landmark. The ‘Weighted-by-Area’ is used for bin-to-cell assignment and Sargent is used for cell type annotation to achieve the highest performance. Supplementary Section S3.5 presents the results for this analysis.

For the Human Colorectal Cancer sample (Supplementary Figs S25 and S26), the top predicted cell types in the ‘muscular’ landmark are Smooth Muscle cells, B cells, and Fibroblasts. In the ‘normal epithelial’ region, the predominant cell types identified are Goblet cells, B cells, and Crypt cells. Within the ‘tumoral epithelium’ region, Enterocytes, Goblet cells, and B cells are most prevalent. Finally, the ‘stromal’ regions are characterized primarily by Fibroblasts, B cells, and Endothelial cells.

In the Mouse Small Intestine sample (Supplementary Figs S27 and S28), the gene markers specified in Supplementary Table ST8 are used by Sargent for cell type annotation. Smooth muscle cells and Paneth cells are the dominant cell types in the ‘muscular’ landmark. In the ‘normal epithelium’ region, Paneth cells, Goblet cells, and B cells are most frequently observed. The ‘lymphoid’ region is predominantly associated with B cells.

Overall, our results demonstrate significant agreement between the predicted cell types and the associated anatomical landmarks, aligning with established biological knowledge of these tissues (Rao and Wang 2010; Kinchen *et al.* 2018; Ghorbaninejad *et al.* 2023). In Supplementary Section S3.5 we present additional results for the application of ENACT to three additional tissues: lung cancer, breast cancer, and tonsil (Supplementary Figs S29–S34).

### 4.3 Comparison of ENACT and Bin2cell

As both ENACT and Bin2cell target Visium HD, we compared their performance using the expert cell annotations (Supplementary Table ST10). Supplementary Section S4 discusses the main differences between ENACT and Bin2cell, as well as the influence of normalization and nuclei expansion on ENACT’s overall performance. As the table shows, ENACT outperforms Bin2cell in the test datasets, especially when using Sargent.

## 5 Discussion

ENACT is a self-contained, tissue-agnostic pipeline designed to streamline the analysis of Visium HD data. ENACT enables users to perform essential initial steps, providing a broad understanding of the tissue’s cellular landscape before progressing to more specialized downstream analyses. Specifically, ENACT allows users to (i) segment cells, (ii) obtain cell-wise transcript counts, (iii) apply one of three available cell annotation methods, (iv) generate visualization-ready files for TissUUmaps, and (v) produce scverse-standard AnnData objects compatible with tools like SquidPy for various spatial statistical analyses, including cell neighborhood enrichment, co-occurrence analysis, and Moran’s I. These features enable ENACT to act as a bridge between initial processing and downstream packages, streamlining the entire analysis pipeline.

When evaluated using expert-annotated data, ENACT achieves an F1-score of 0.787 and an accuracy of 0.761, an absolute improvement of 0.170 in F1-score over prior pipelines, demonstrating the advantages of splitting the transcript content of overlapping bins (Supplementary Table ST10). On synthetic data, the weighted methods exhibit superior overall precision and recall compared to the ‘Naive’ method. Overall, the ‘Weighted-by-Area’ method, when paired with the Sargent cell annotation method, delivers the best performance. Supplementary Section S4.2 highlights that incorporating nuclei expansion further enhances the accuracy of cell-type annotations.

ENACT has been applied to a variety of public datasets spanning two species (mouse and human), five distinct tissues (lung, tonsil, breast, small intestine, and colon), and two sample types (FFPE and fresh frozen), showcasing its tissue-agnostic capabilities. These datasets and their corresponding results are detailed in Supplementary Sections S2.3 and S3.5, respectively. ENACT’s performance, and the selection of bin assignment methods, is influenced by factors such as cell segmentation quality, cell density, transcript sparsity, cell granularity, and gene marker selection. These factors are discussed in detail in Supplementary Section S5 to provide guidance for users.

## Supplementary Material

btaf094_Supplementary_Data
