# Supplementary material for: ENACT: End-to-End Analysis of Visium High Definition (HD) Data
Source: Bioinformatics. 2025 Mar 7;41(3):btaf094. doi: 10.1093/bioinformatics/btaf094 (PMC11925495; doi:10.1093/bioinformatics/btaf094)
Supplement: btaf094_Supplementary_Data [file btaf094_supplementary_data.zip › supplementary material - feb 24 - small.pdf]

Bioinformatics, 2024, 1–37
doi: DOI HERE
Supplementary Material

ENACT: End-to-End Analysis and Cell Type Annotation for Visium High Definition (HD) Slides
Supplementary Material

Mena Kamel,[1, †] Yiwen Song,[1, †] Ana Solbas,[4] Sergio Villordo,[4] Amrut Sarangi,[1] Pavel Senin,[1] Sunaal Mathew,[1] Luis Cano Ayestas,[2] Clement Levin,[2] Seqian Wang,[1] Marion Classe,[2] Ziv Bar-Joseph[3,*] and Albert Pla Planas[4,*]

[1]Digital R&D, Sanofi, Toronto, ON, Canada, [2]Precision Medicine & Computational Biology, Sanofi, Paris, France, [3]Digital R&D, Sanofi, Cambridge, MA, USA and [4]Digital R&D, Sanofi, Barcelona, Spain

[†]Equal contribution [*]Corresponding authors. ziv.bar-joseph@sanofi.com, albert.plaplanas@sanofi.com

Abstract

This document provides the supplementary material for ENACT. ENACT is a self-contained pipeline designed to streamline Visium HD analysis from cell segmentation to annotation, enabling integration with advanced spatial analysis tools. ENACT enables users to perform essential initial steps, providing a broad understanding of the tissue's cellular landscape before moving on to more specialized downstream analyses. In particular, ENACT allows users to (1) segment cells, (2) obtain cell-wise transcript counts, (3) apply one of three available cell annotation methods, (4) generate visualization-ready files for TissUUmaps, and (5) produce AnnData objects compatible with tools like SquidPy. By adhering to the scverse-standard AnnData format, ENACT outputs can be easily integrated into various spatial statistical analyses, such as cell neighborhood enrichment, co-occurrence analysis, and Moran's I. These capabilities empower users to seamlessly connect ENACT with downstream packages, thereby streamlining the overall analysis. Code, installation instructions and examples are publicly available on GitHub at `https://github.com/Sanofi-Public/enact-pipeline`. Experimental data is available at `https://zenodo.org/records/14748859`.

1. Bin-to-Cell Assignment Methods

Bin-to-cell assignment consists of assigning the transcripts present in VisiumHD bins to the cells segmented in the H&E image. Given the bin size of 2μm, most bins completely overlap a single cell; however, some bins may partially cover a cell or overlap multiple cells.

Bin-to-cell assignment begins by omitting the Visium HD bins that do not geometrically intersect any cell outlines as described in Figure 1. Let B denote the set of bins overlapping cells, we can separate them in bins that overlap a unique cell (unique bins, B_{unique}) and in bins that overlap multiple cells (shared bins, B_{shared}).

$$B = B_{\text{unique}} \cup B_{\text{shared}} \quad (1)$$

© The Author 2024. Published by Oxford University Press. All rights reserved. For permissions, please e-mail: journals.permissions@oup.com

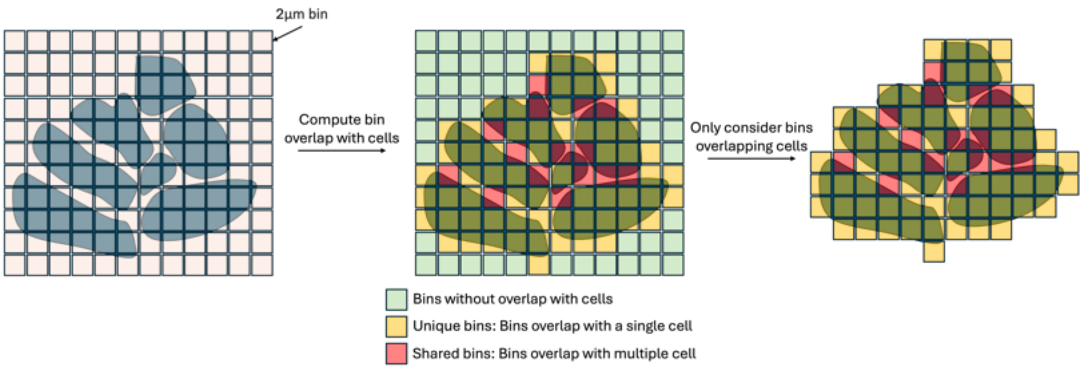

Fig. 1: Bins are classified into three types: (1) bins that do not overlap with any cells (green), (2) 'unique bins': bins that overlap with a single cell (yellow), and (3) 'shared bins': bins that overlap multiple cells (red). The weighted methods are designed to split the transcripts within the 'shared bins' across the cells that overlap them.

Using the 2 μm bins provided by Visium HD, ENACT proposes the following different strategies for bin-to-cell assignment:

1.1. Naive method

This method only uses the B_{unique} set. Bins that intersect multiple cells (B_{shared}) are omitted (SF 2(a)).

$$\mathrm{G}_{C_j} = \sum_{i=1}^{N} \alpha_i \times G_{B_i}, \quad \alpha_i = \begin{cases} 1 & \text{if } i \in B_{\text{unique}} \\ 0 & \text{if } i \in B_{\text{shared}} \end{cases} \tag{2}$$

Here, G_{C_j} are the transcript counts assigned to cell C_j and G_{B_i} represents the transcript counts within bin B_i. N is the total number of bins that intersect C_j. α_i is a weighting factor used to dictate the contribution of bin B_i to cell C_j. α_i is set to zero for the shared bins to discard their contributions to the total cell counts in the naive method. The final cell transcript counts are obtained by summing the contribution from all the unique bins that overlap with the corresponding cell (SF 2(b, c)).

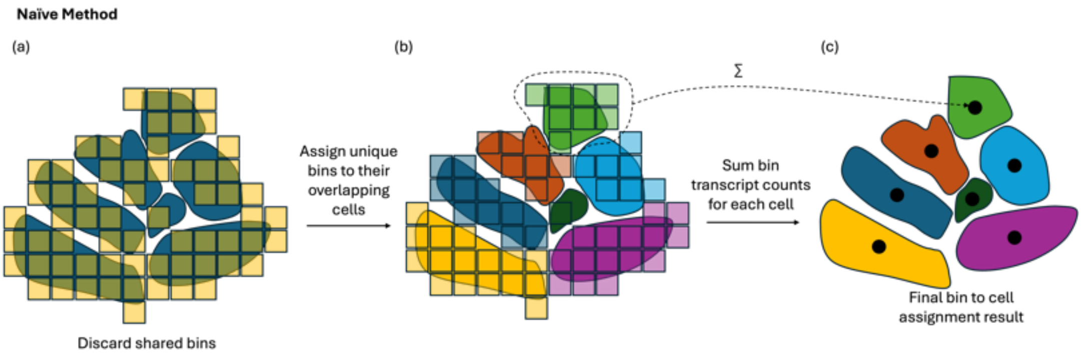

Fig. 2: (a) Naive method only considers bins containing a unique cell (*unique bins*) and discards the bins with more than one (*shared bins*). (b) Unique bins are assigned to the cells they overlap with. (c) Final cell transcript counts is the sum of the contribution from all the unique bins that overlap the corresponding cell.

1.2. Weighted-by-Area

The 'Naive' method leads to information loss since all the shared bins are discarded. The Weighted-by-Area approach proposes to assign bins in B_{shared} to cells they overlap by weighting the contribution of the bin to the cell based on the area of overlap, $\text{Area}_{C_j \cap B_i}$. Like the naive method, the weighting factor α_i is set to 1 for all the unique bins in B_{unique}. For the bins in B_{shared}, α_i is the ratio of the bin's overlap area with the cell to the total bin area as described in Eq. 3 and SF 3.

$$\mathrm{G}_{C_j} = \sum_{i=1}^{N} \alpha_i \times G_{B_i}, \quad \alpha_i = \begin{cases} 1 & \text{if } i \in B_{\text{unique}} \\ \frac{\text{Area}_{C_j \cap B_i}}{\text{Area}_{B_i}} & \text{if } i \in B_{\text{shared}} \end{cases} \tag{3}$$

Here, Area_{B_i} is the total 2 μm bin area ($\sim 4\ \mu\text{m}^2$). Area_{C_j} is the intersection area between cell C_j and bin B_i.

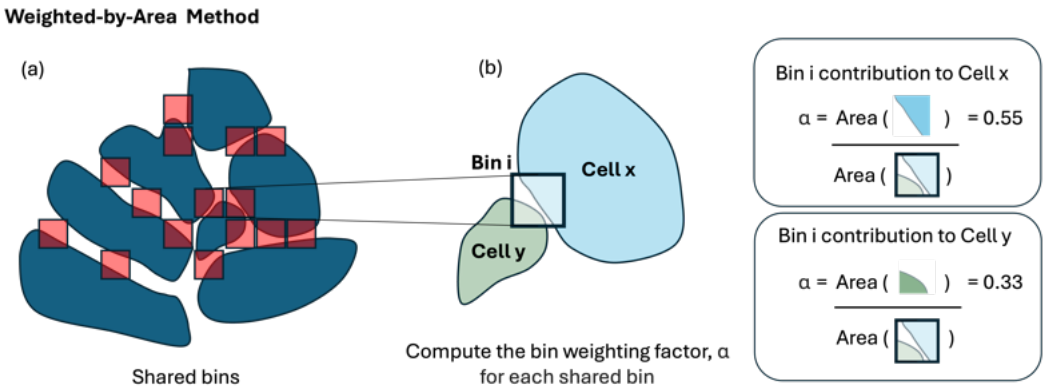

Fig. 3: (a) The Weighted-by-Area method assigns weights to shared bins based on the overlap area with individual cells. (b) For each cell, the weight α_i of a bin in B_{shared} is calculated as the ratio of the bin's overlap area with the cell to the total bin area ($\sim 4\ \mu\text{m}^2$).

1.3. Weighted-by-Transcript

While the Weighted-by-Area method allows the assignment of all overlapping bins, such an assignment ignores the fact that neighboring cells can be very different. Thus, simply dividing the transcripts based on area may not provide the correct assignment of these transcripts. This method attempts to address this issue by weighting transcript counts based on their expression in the cells overlapping the bin.

Weighted-by-Transcript uses the naive assignment to determine the transcripts in each cell's unique bins. Then it uses the preliminary transcript distribution of each cell to estimate the (α) of transcript reads in shared bins to each of its overlapping cells. α is computed separately for each of the T genes based on their expression in the surrounding cells (SF 4(b)):

$$\begin{gathered} \mathrm{G}_{C_j} = \sum_{i=1}^{N} \left(\sum_{g=1}^{T} \alpha_{i,g} \times G_{B_i,g} \right), \\ \alpha_{i,g} = \begin{cases} 1 & \text{if } i \in B_{\text{unique}} \\ \frac{\hat{G}_{C_j,g}}{\sum_{s=1}^{n} \hat{G}_{C_s,g}} & \text{if } i \in B_{\text{shared}} \end{cases} \end{gathered} \tag{4}$$

where:

- T is the total number of unique genes,
- $\alpha_{i,g}$ is the weighting for gene g in bin i,
- $G_{B_i,g}$ is the count of gene g in bin B_i,
- $\hat{G}_{C_j,g}$ is the normalized count of gene g in cell C_j, and
- n is the number of cells that overlap with bin B_i.

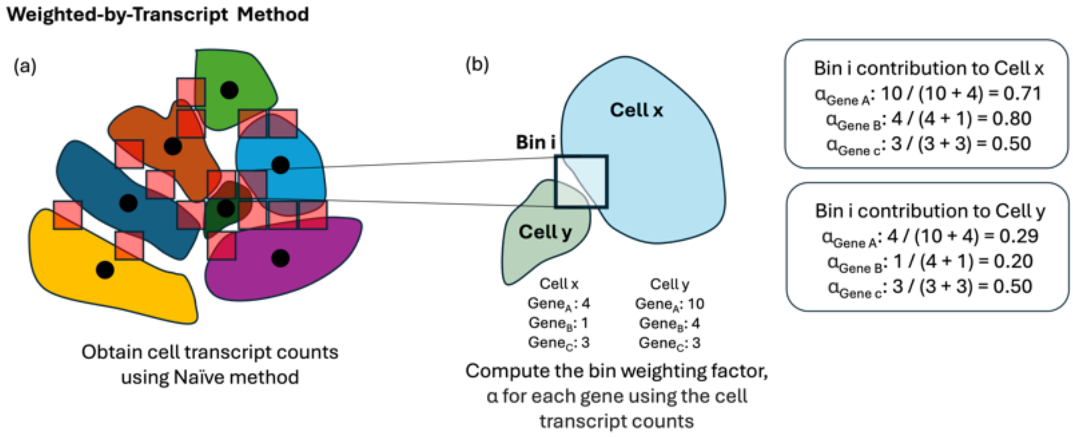

Fig. 4: (a) The Weighted-by-Transcript method begins by applying the Naive method to estimate preliminary transcript counts for each cell using bins in B_{unique}. (b) For each cell j, the weight $\alpha_{i,g}$ assigned to bin i for gene g is calculated as the proportion of the normalized transcript count of gene g in cell j compared to the total normalized transcript count of the same gene in all *other* cells sharing bin i.

1.4. Weighted-by-Cluster

One problem with the Weighted-by-Transcript method is that if an overlapping bin contains a gene that has not been expressed in any of the other bins of the intersecting cells, the weighting factor $\alpha_{i,g}$ would be 0 for all cells leading to information loss. This may impact the assignment of many genes expressed at low levels which can still play an important role in the process being studied (Rizzetto et al. 2017). To address this, here α is computed by using the (average) expression in similar cells. Specifically, we first use the 'Naive' method to obtain individual cell transcript estimates. Then, K-means clustering is used to group cells based on gene expression. For each cluster, the average transcript counts are computed and used for assigning the remaining bins.

$$\begin{aligned}
\mathrm{G}_{C_j} &= \sum_{i=1}^{N} \left(\sum_{g=1}^{T} \alpha_{i,g} \times G_{B_i,g} \right), \\
\alpha_{i,g} &= \begin{cases} 1 & \text{if } i \in B_{\text{unique}} \\ \frac{\hat{G}_{\mathcal{K}(C_j),g}}{\sum_{s=1}^{n} \hat{G}_{\mathcal{K}(C_s),g}} & \text{if } i \in B_{\text{shared}} \end{cases} \\
\mathcal{K}(C) &= \text{K-means}(C)
\end{aligned} \tag{5}$$

where $\mathcal{K}(C)$ is the cluster that cell C is assigned to. SF 5 describes the Weighted-by-Cluster method in detail.

It is worth noting that while this method may increase the accuracy of transcript assignment, the additional clustering step increases the computational complexity of the assignment and may result in longer processing times (see Supplementary Section 3.3).

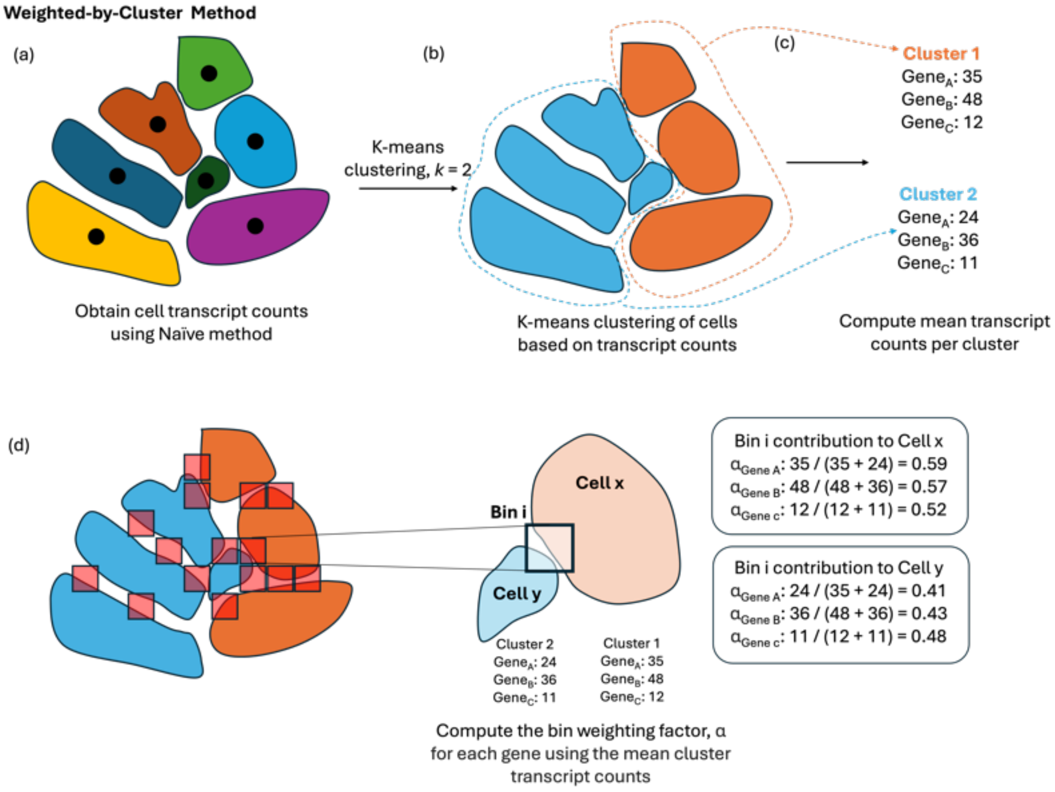

Fig. 5: (a) The Weighted-by-Cluster method builds on the Weighted-by-Transcript method by grouping cells into k clusters based on their preliminary transcript counts, which are estimated using the Naive method and bins in B_{unique}. (b) K-means clustering is used to assign cells to clusters, and the mean transcript count for each gene is computed per cluster. (c) For each cell j, the weight $\alpha_{i,g}$ assigned to bin i for gene g is calculated using the normalized transcript count of gene g in the cluster containing cell j, rather than the absolute transcript count of the cell itself. This weight is determined as the ratio of the cluster's normalized transcript count of gene g to the total normalized cluster transcript count of the same gene across all *other* cells sharing bin i.

2. Evaluation Datasets

2.1. Datasets for Evaluating Bin-to-Cell Assignment Methods

To evaluate the accuracy of transcript assignment, two synthetic Visium HD-like datasets are constructed. The first dataset is constructed from Xenium datasets provided by 10x Genomics, profiling FFPE Human Colorectal Cancer (Xenium demo data[1]). This dataset includes 386,695 segmented cells/nuclei and 545 distinct genes. As Xenium is an imaging-based technology that provides pinpoint locations of transcripts within the tissue, it serves as a good baseline for assessing whether our bin-to-cell methods accurately map transcript locations.

The second dataset is from sequential fluorescence in situ hybridization (seqFISH+). The seqFISH+ dataset (Eng and Cai 2019) contains spatial mRNA data for 10,000 genes, offering gene coverage that is more akin to Visium HD. Like Xenium, this is also an imaging method making it easier to assign specific transcripts to cells. The dataset contains data from 103 mouse embryonic fibroblast (NIH-3T3) where cells were manually segmented[2].

To generate synthetic datasets from these two datasets we artificially assign a 2µm x 2µm grid to the image and summed up the transcript abundance in each bin as described in Figure 6. Additionally, since our segmentation model, Stardist, focuses on nuclei segmentation and the Xenium dataset provides information on both nuclei and whole cell boundaries, we assess the assignment methods by comparing their performance in assigning transcripts to both nuclei and whole cell boundaries.

The datasets are statistically described in Figures 7-12 in terms of distribution and transcript density both at cellular and bin level as well as the distribution of unique and shared bins. The cell nearest neighbor distance is calculated as the Euclidean distance between cell centroids, while cell density is determined by counting the number of cells within 1000 μm^2 patches.

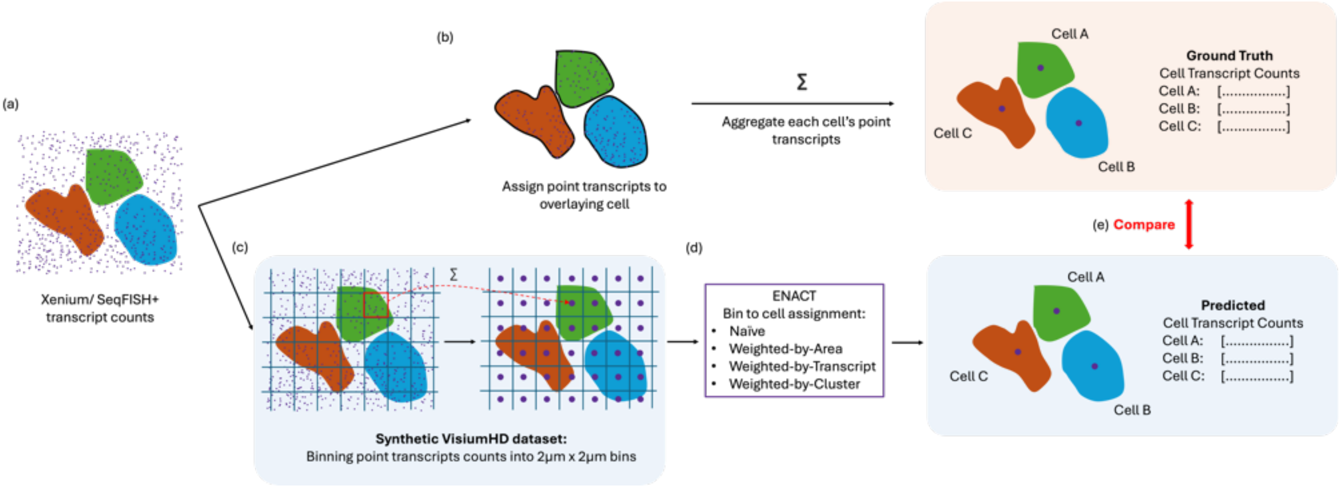

Fig. 6: Synthetic Visium HD dataset generation and bin-to-cell assignment evaluation process. (a) Xenium and SeqFISH+ public datasets consisting of point-based transcripts, their locations, and cell outlines. (b) Ground truth cell transcript counts are obtained by assigning the point-based transcript counts to their overlaying cell outlines. Cell-specific transcript counts are aggregated (summed) to obtain the ground truth cell transcript counts. (c) Synthetic Visium HD dataset is constructed by grouping the point transcript counts into virtual 2µm x 2µm bins and aggregating (summing) the transcript counts within each synthetic bin. (d) ENACT bin-to-cell assignment step is run on the synthetic Visium HD dataset to obtain the 'predicted' cell transcript counts. (e) Each bin-to-cell assignment method is evaluated relative to the ground truth cell transcript counts. This process is repeated for both Xenium and SeqFISH-based synthetic datasets.

[1] https://www.10xgenomics.com/datasets/ffpe-human-colorectal-cancer-data-with-human-immuno-oncology-profiling-panel-and-custom-add-on-1-standard

[2] https://zenodo.org/records/2669683#.Xqi1w5NKg6g

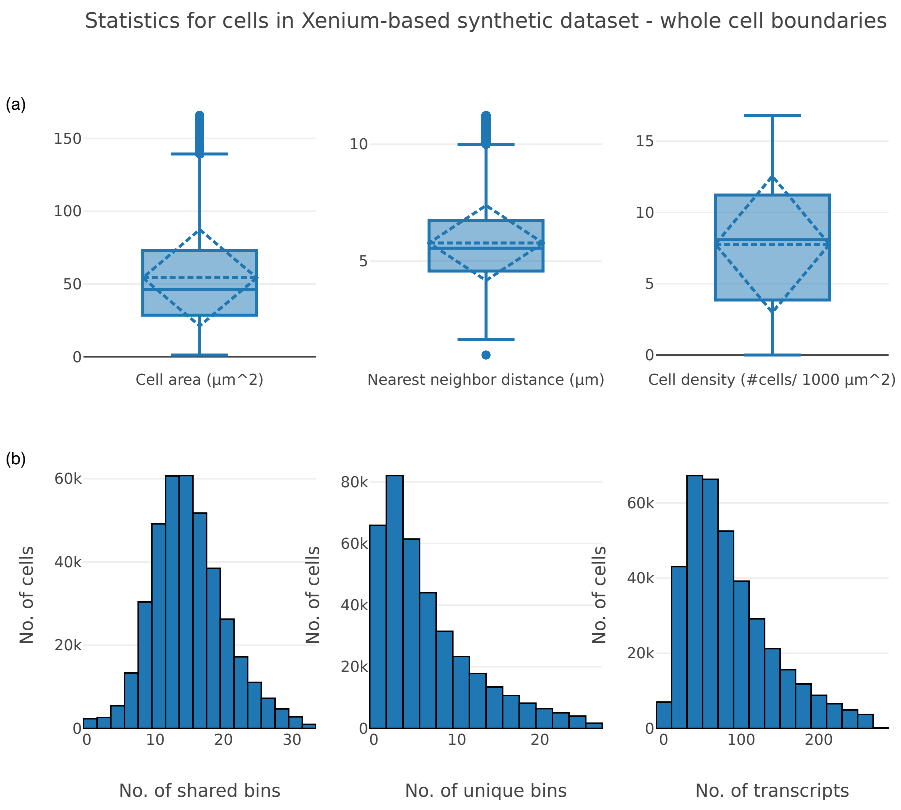

Fig. 7: Cell statistics for the Xenium-based Visium HD dataset, considering whole cell boundaries. (a) Distributions of cell area, nearest neighbor distance, and cell density. Except for cell density, where each point represents a 1000 μm^2 patch, each data point corresponds to a single cell. (b) Distributions of the number of shared and unique bins, and the number of transcripts per cell after applying ENACT's bin-to-cell assignment step using the Weighted-by-Area method.

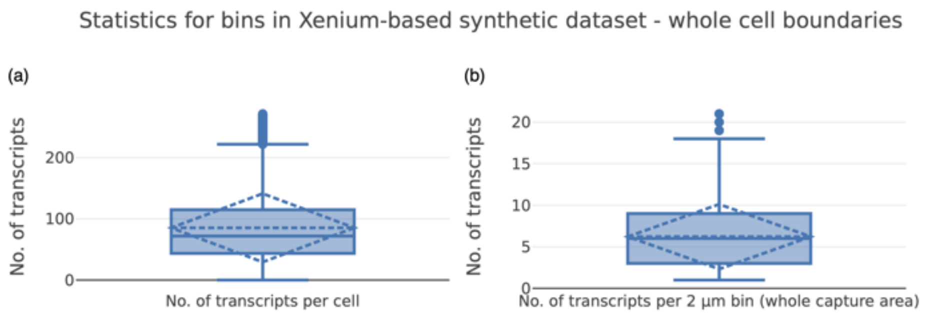

Fig. 8: Bin statistics for the Xenium-based Visium HD dataset, considering whole cell boundaries. (a) Distribution of the number of transcripts per cell after applying ENACT's bin-to-cell assignment step using the Weighted-by-Area method. (b) Distribution of the number of transcripts per 2µm. Here, each data point represents a cell.

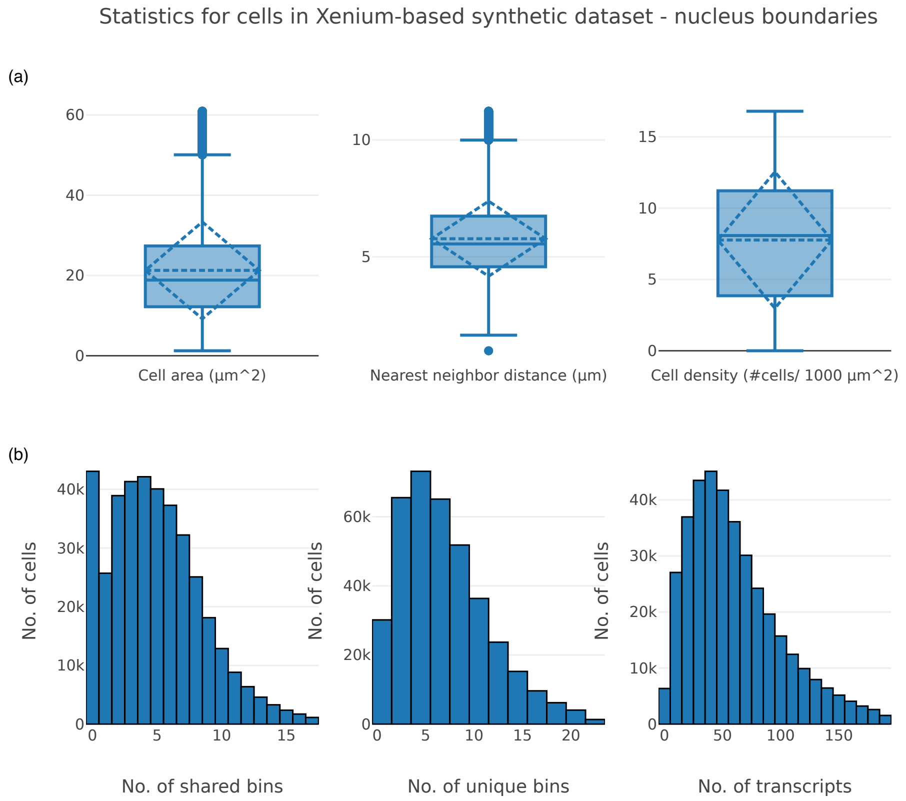

Fig. 9: Cell statistics for the Xenium-based Visium HD dataset, considering nuclei boundaries. (a) Distributions of cell area, nearest neighbor distance, and cell density. Except for cell density, where each point represents a 1000 μm^2 patch, each data point corresponds to a single cell. (b) Distributions of the number of shared and unique bins, and the number of transcripts per cell after applying ENACT's bin-to-cell assignment step using the Weighted-by-Area method.

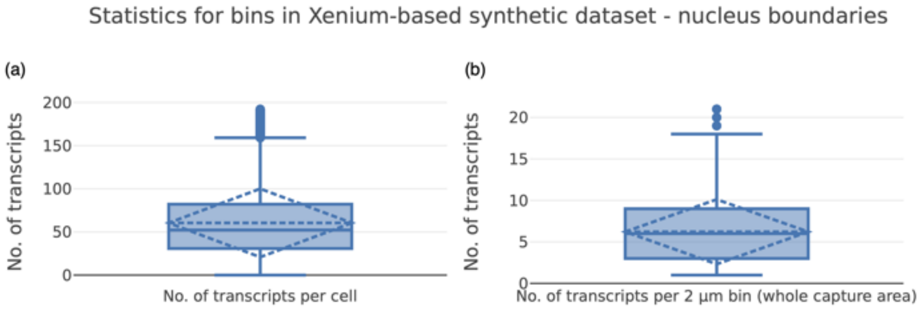

Fig. 10: Bin statistics for the Xenium-based Visium HD dataset, considering nuclei boundaries. (a) Distribution of the number of transcripts per cell after applying ENACT's bin-to-cell assignment step using the Weighted-by-Area method. (b) Distribution of the number of transcripts per 2µm. Here, each data point represents a cell.

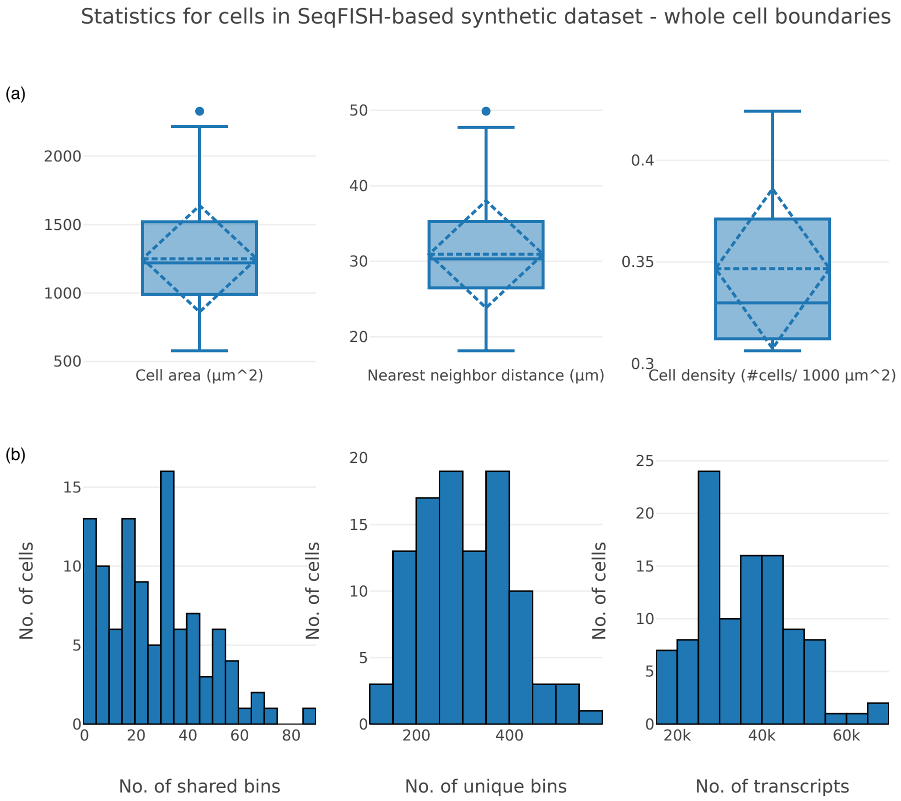

Fig. 11: Cell statistics for the SeqFISH-based Visium HD dataset, considering whole cell boundaries. (a) Distributions of cell area, nearest neighbor distance, and cell density. Except for cell density, where each point represents a 1000 μm^2 patch, each data point corresponds to a single cell. (b) Distributions of the number of shared and unique bins, and the number of transcripts per cell after applying ENACT's bin-to-cell assignment step using the Weighted-by-Area method.

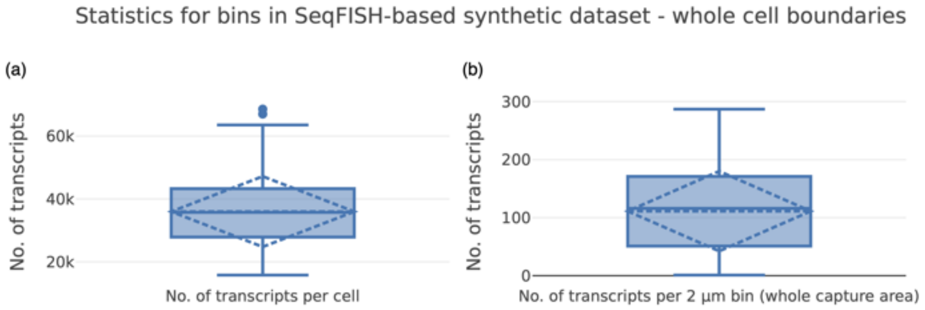

Fig. 12: Bin statistics for the SeqFISH-based Visium HD dataset, considering whole cell boundaries. (a) Distribution of the number of transcripts per cell after applying ENACT's bin-to-cell assignment step using the Weighted-by-Area method. (b) Distribution of the number of transcripts per 2µm. Here, each data point represents a cell.

2.2. Datasets for Evaluating Cell-Type Annotation

To validate the end-to-end pipeline, five publicly available FFPE and Fresh Frozen Visium HD samples were annotated by experts and then analyzed using ENACT, demonstrating ENACT as a tissue-agnostic pipeline:

- **Human Colorectal Cancer FFPE**[3] sample from the sigmoid of a 60 year old male subject,
- **Mouse Small Intestine FFPE**[4] sample from 8-week old male mouse,
- **Human Lung CancerFFPE**[5] sample from a subject with Adenocarcinoma (age and gender unspecified),
- **Human Tonsil Fresh Frozen**[6] sample from a 21 year old male subject with Reactive Follicular Hyperplasia,
- **Human Breast Cancer Fresh Frozen**[7] sample from a 58 year old female subject with Ductal Carcinoma in Situ (DCIS).

All of these samples are captured using Visium CytAssist with Visium Human Transcriptome Probe Set (v2.0) and Visium Mouse Transcriptome Probe Set (v2.0). The results from these samples are presented in subsection 3.5. For each of these samples, annotations are obtained in the form of anatomical landmarks (labelled tissue regions) and manually annotated cell type labels (colorectal cancer sample only), as shown in Figure 13, Figure 30(a), Figure 32(a), amd Figure 34(a). These annotations are provided by a pathologist and an immunologist based solely on the corresponding histopathology H&E tissue images. Table 1 presents the breakdown of the different anatomical landmarks labeled for each sample.

Table 2 shows the breakdown of the 20,991 manually curated cell type labels obtained from four tissue patches in the Human Colorectal Cancer sample. ENACT is run on the Human Colorectal cancer sample to obtain cell-type labels and the results are compared to the ground truth expert annotations. Figures 14-17 characterize the cell areas, tissue sparsity, nearest neighbor distance, numbers of shared and unique bins, as well as transcript abundance per cell, per bin, and per cell type predicted by ENACT.

Sample Type	Tissue Landmarks
Human Colorectal Cancer	Muscular, Normal Epithelium, Tumoral Epithelium, Stroma
Mouse Small Intestine	Lymphoid Tissue, Muscular, Normal Epithelium
Human Lung Cancer	Anthracosis, Blood Vessel, Immune Cells, Normal Bronchial Smooth Muscle, Normal Bronchial Epithelium, Normal Lung Parenchyma, Red Blood Cells, Tumor
Human Tonsil	Lymphoid Tissue
Human Breast Cancer	Stroma, Tumor

Table 1. List of sample types and their corresponding tissue anatomical landmarks.

Cell Type	Number of Annotated Cells
Epithelial cells	12072
Stromal cells	6171
Immune cells	2748

Table 2. List of cell types and their corresponding count in the evaluation dataset

[3] https://www.10xgenomics.com/datasets/visium-hd-cytassist-gene-expression-libraries-of-human-crc
[4] https://www.10xgenomics.com/datasets/visium-hd-cytassist-gene-expression-libraries-of-mouse-intestine
[5] https://www.10xgenomics.com/datasets/visium-hd-cytassist-gene-expression-human-lung-cancer-post-xenium-expt
[6] https://www.10xgenomics.com/datasets/visium-hd-cytassist-gene-expression-human-tonsil-fresh-frozen
[7] https://www.10xgenomics.com/datasets/visium-hd-cytassist-gene-expression-human-breast-cancer-fresh-frozen

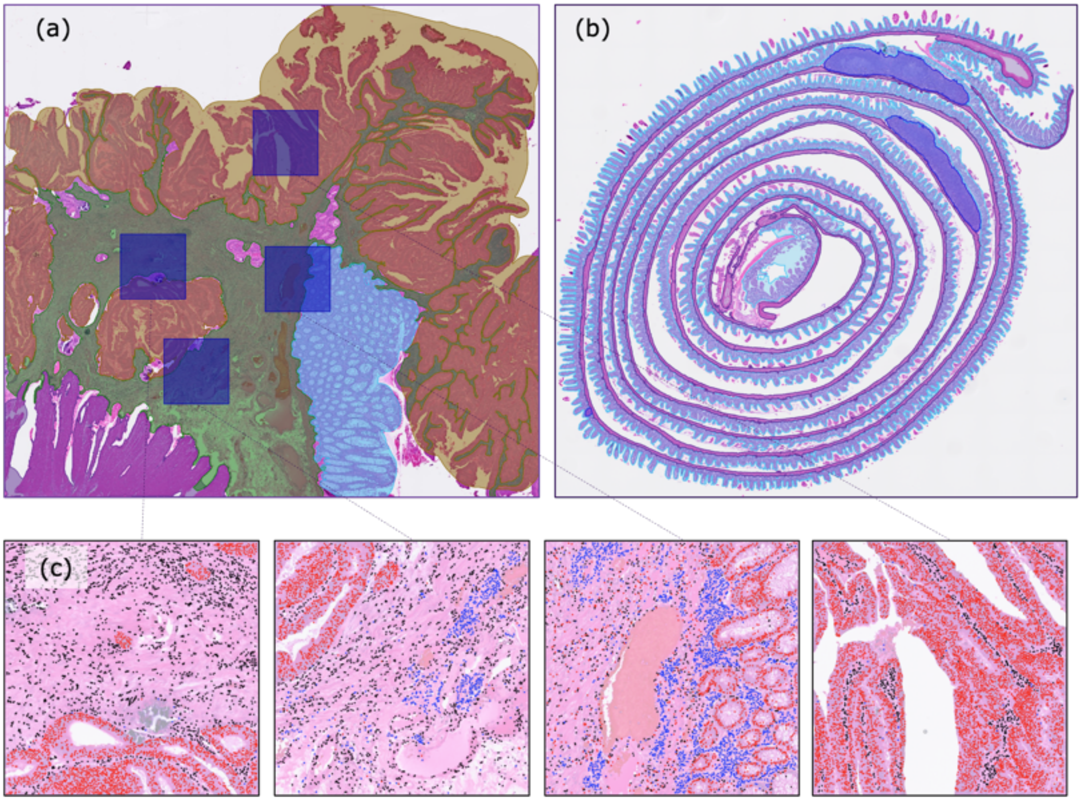

Fig. 13: (a) Human Colorectal cancer sample with pathologist-annotated anatomical landmarks for Tumoral Epithelium (yellow), Normal Epithelium (blue), Muscular (purple), and Stroma (green) areas. (b) Mouse small intestine sample with pathologist-annotated anatomical landmarks for Lymphoid Tissue (purple), Muscular (pink), Normal Epithelium (blue) areas. (c) Patches from Human Colorectal cancer sample with individually annotated cell labels for Epithelial cells (red), Immune cells (blue), and Stromal cells (black).

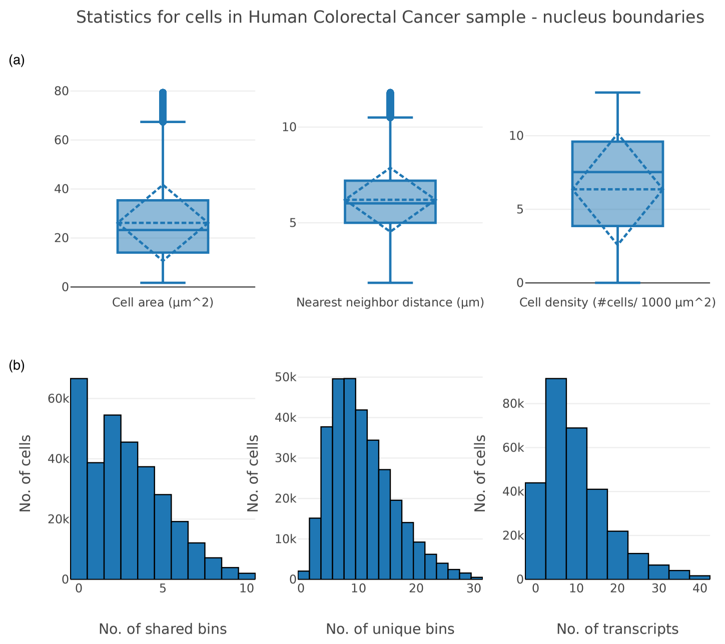

Fig. 14. Cell statistics for the human colorectal cancer sample, considering nuclei boundaries. Statistics presented here are based on cells segmented and analyzed by ENACT using the Weighted-by-Area method. (a) Distributions of cell area, nearest neighbor distance, and cell density. Except for cell density, where each point represents a $1000\ \mu m^2$ patch, each data point corresponds to a single cell. (b) Distributions of the number of shared and unique bins, and the number of transcripts per cell after applying ENACT's bin-to-cell assignment step using the Weighted-by-Area method. Note: number of transcripts per cell only includes the subset of top-1000 highly variable genes.

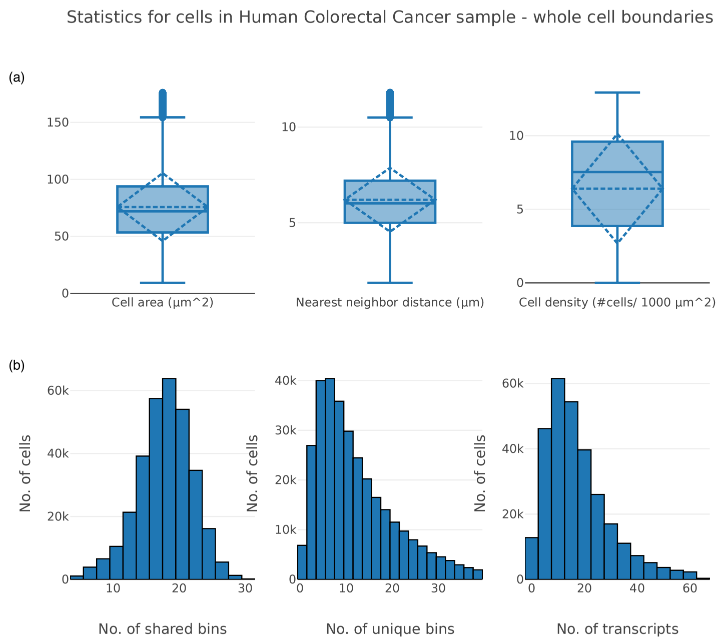

Fig. 15. Cell statistics for the human colorectal cancer sample, considering whole cell boundaries. Statistics presented here are based on cells segmented and analyzed by ENACT using the Weighted-by-Area method. (a) Distributions of cell area, nearest neighbor distance, and cell density. Except for cell density, where each point represents a $1000\ \mu m^2$ patch, each data point corresponds to a single cell. (b) Distributions of the number of shared and unique bins, and the number of transcripts per cell after applying ENACT's bin-to-cell assignment step using the Weighted-by-Area method. Note: number of transcripts per cell only includes the subset of top-1000 highly variable genes.

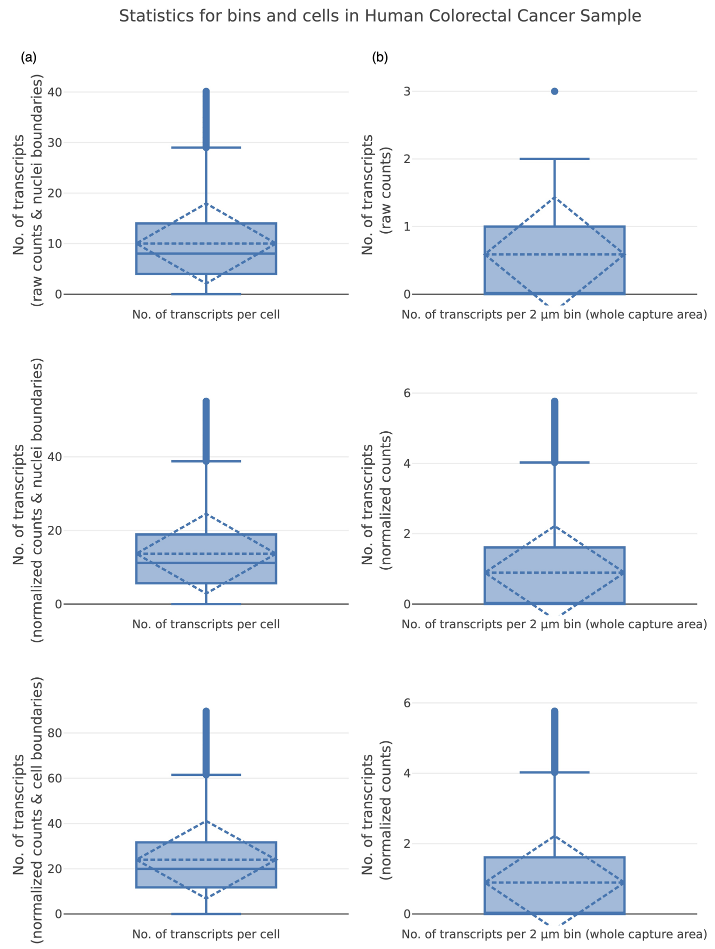

Fig. 16. (a) Raw and normalized transcript counts per cell when considering nuclei and cell boundaries. (b) Raw and normalized transcript counts per bin. Transcript counts only include the subset of top-1000 highly variable genes.

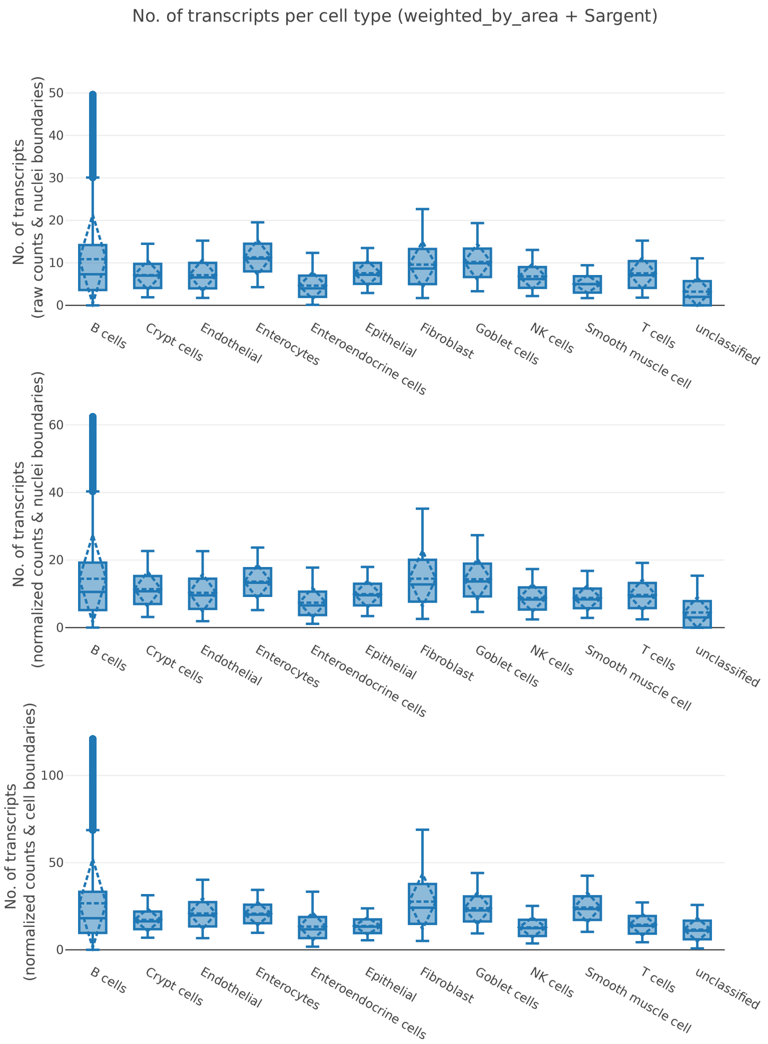

Fig. 17. Raw and normalized transcript counts per cell type when considering nuclei and cell boundaries. Transcript counts only include the subset of top-1000 highly variable genes.

3. Results

3.1. Evaluating Bin-to-Cell Assignment Methods

This section describes the performance of the different bin-to-cell assignment methods on the Xenium- and (SeqFISH+)- based synthetic datasets. For the Xenium-based dataset, both the whole cell and nuclei boundaries are used for bin-to-cell assignment. For the SeqFISH+ dataset, only whole cell boundaries are considered since the nuclei boundaries are not provided.

3.1.1. Performance on Xenium-Based Synthetic Dataset

Figure 18 and Figure 19 show violin plots of the results from running ENACT on the Xenium-based synthetic Visium HD datasets, considering whole cell boundaries and nuclei boundaries, respectively. The dataset is divided into patches of size 1000x1000 pixels, each evaluated individually against the ground truth bin-to-cell assignment data (Figure 6(b)) and presented as a data point in the violin plots.

Method	Precision Avg.	Recall Avg.	F1 Avg.
Naive	**0.98**	0.48	0.65
Weighted-by-Area	0.90	**0.87**	**0.88**
Weighted-by-Transcript	0.82	0.70	0.76
Weighted-by-Cluster	0.812	0.83	0.82

Table 3. Evaluation of the different bin-to-cell assignment methods on the Xenium-based synthetic dataset using the whole cell boundaries.

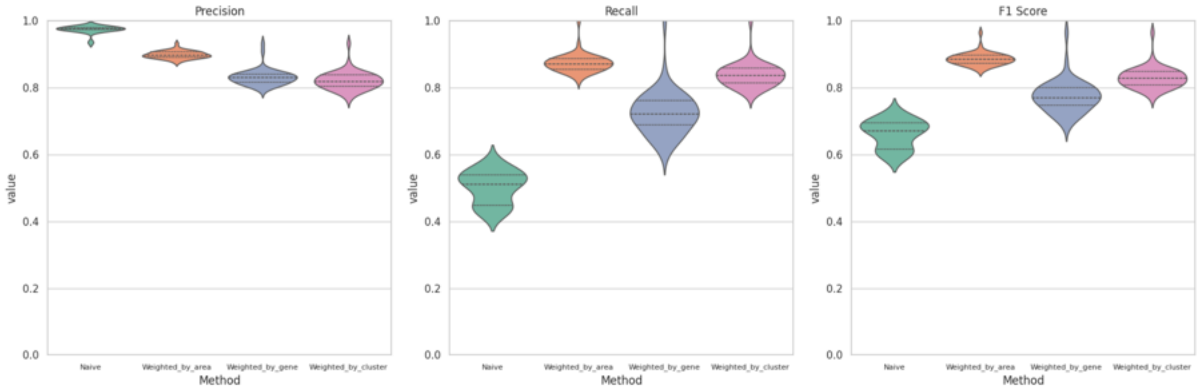

Fig. 18: Violin plots of the precision, recall, and F1 score of the four bin-to-cell assignment methods evaluated on the Xenium-based synthetic dataset using the whole cell boundaries.

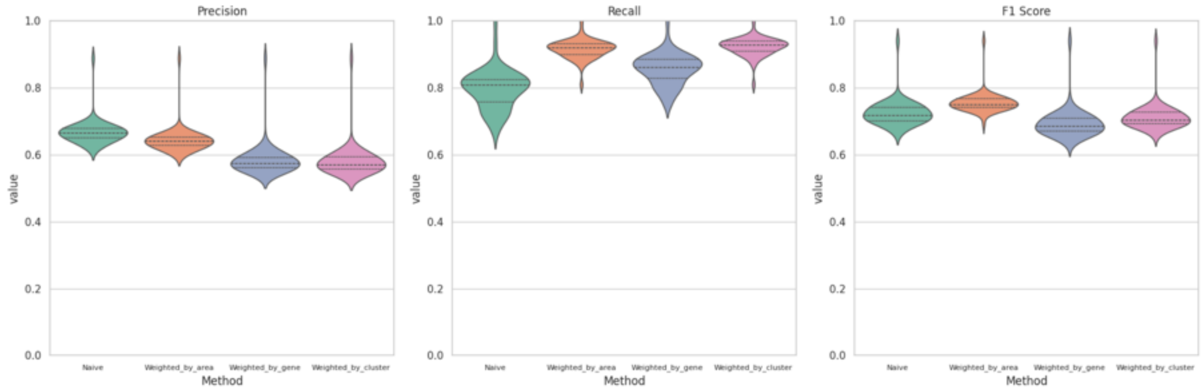

Fig. 19: Violin plots of the precision, recall, and F1 score of the four bin-to-cell assignment methods evaluated on the Xenium-based synthetic dataset using the nuclei boundaries.

3.1.2. Performance on (seqFISH+)-Based Synthetic Dataset

Figure 20 shows violin plots of the results from running ENACT on the (SeqFISH+)-based synthetic Visium HD datasets, considering whole cell boundaries. The dataset is divided into patches of size 1000x1000 pixels, each evaluated individually against the ground truth bin-to-cell assignment data (Figure 6(b)) and presented as a data point in the violin plots.

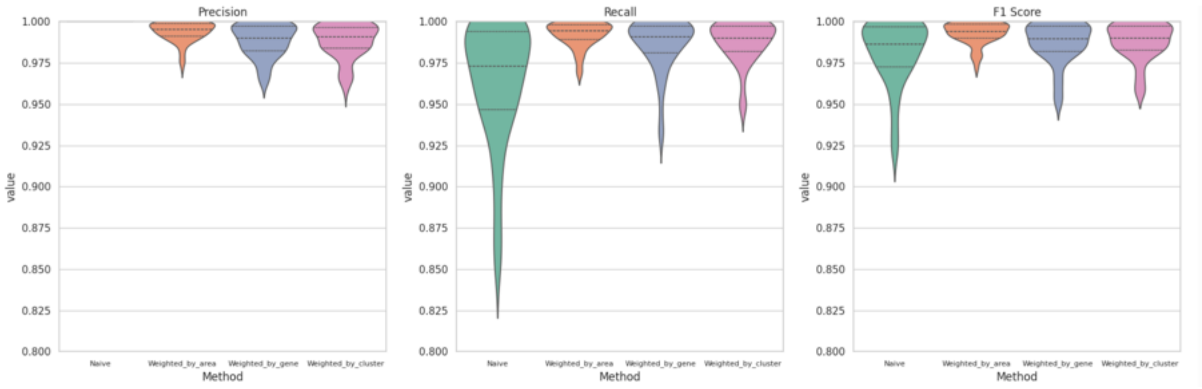

Fig. 20: Violin plots of the precision, recall, and F score of the four bin-to-cell assignment methods evaluated on the (SeqFISH+)-based synthetic dataset using the whole cell boundaries.

3.2. Analyzing Effect of Number of Clusters on Weight-by-Cluster

The number of clusters used in the Weight-By-Cluster assignment method, k (default $k = 4$), can influence bin-to-cell assignment and predicted cell types. Since Weighted-by-Cluster is memory intensive, it is not run in the whole slide but iteratively on smaller patches (dictated by the 'patch_size' parameter) where cell type variability is lower, making the default k = 4 a reasonable choice for a patch of size 4000x4000 pixels. Figure 21 shows that increasing k from 2 to 5 results in marginal improvements in precision, recall, and F1-score (0.5%, 0.6%, and 0.4%, respectively), with a slight drop for $k > 5$. Larger patch sizes may increase cell type variability, where higher k values could be considered.

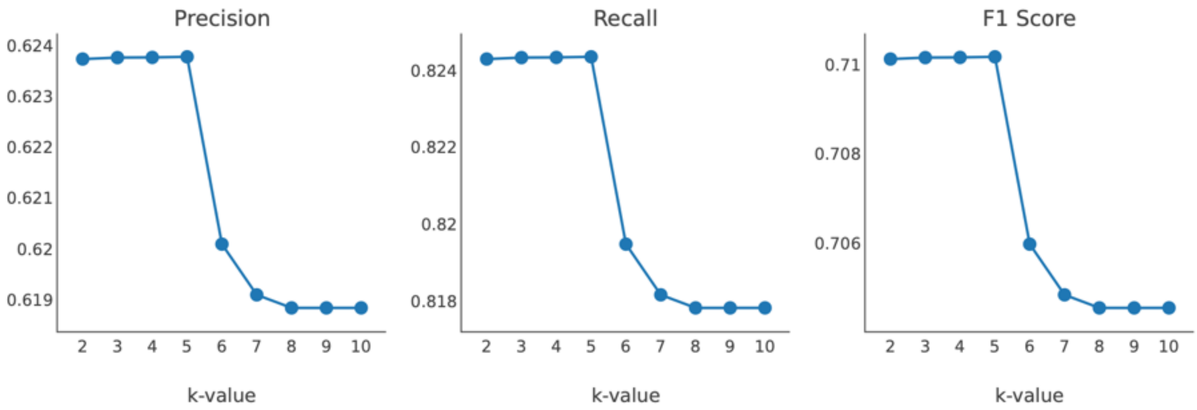

Fig. 21: Performance of Weighted-by-Cluster method relative to ground truth Xenium-based synthetic Visium HD dataset. Here, analysis is done on a 3000 x 3000 pixel patch of the synthetic dataset. Nuclei boundaries are used. The number of principal components for K-means clustering is set to 250.0

3.3. Bin-to-Cell Assignment Method Running Time Analysis

The run time for the four bin-to-cell assignment methods is computed by measuring time needed to perform bin-to-cell assignment on patches of size 4000 x 4000 pixels from the Human colorectal cancer public sample [8]. In total, 42 patches are analyzed, each containing around 10,000 cells. Here, only the top-1000 most highly variable genes are used used for the analysis.

	Running Time Avg. (Second)
**Naive**	7.01
**Weighted-by-Area**	15.2
**Weighted-by-Transcript**	61.3
**Weighted-by-Cluster**	78.0

Table 4. This table summarizes the execution time (in seconds) for each bin-to-cell assignment method when processing a 4000 × 4000 pixel image patch with around 10000 cells.

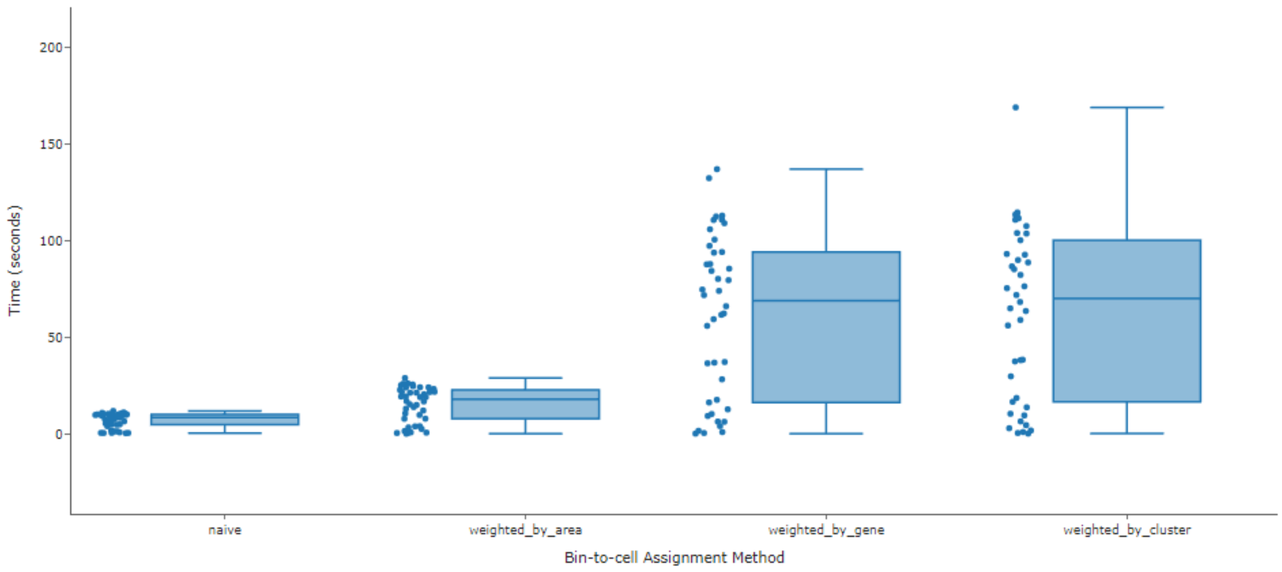

Fig. 22: Execution time (in seconds) for each bin-to-cell assignment method when processing a 4000 x 4000 pixel image patch with around 10000 cells. Here, each blue dot represents the run time for a given patch.

[8] https://www.10xgenomics.com/datasets/visium-hd-cytassist-gene-expression-libraries-of-human-crc

3.4. Comparing Predicted Cell Types to Pathologist Annotations

The predictions from ENACT are compared against pathologist-curated cell labels from the Human colorectal cancer public sample. Here, twelve experiments are conducted to compare the effect of the bin-to-cell assignment method and cell annotation algorithm on the pipeline performance. The top-1000 most highly variable genes in addition to the gene markers defined in Table 7 are used for bin-to-cell assignment and cell annotation. Table 5 describes the mapping between the granular cell labels predicted by Sargent, CellAssign, and CellTypist and the pathologist-provided cell label. Table Table 10 provides the summary of the evaluation in terms of accuracy, precision, recall, and F1-score. Here, the four proposed bin-to-cell assignment methods are run using the nuclei boundaries predicted by Stardist and the raw (non-normalized) transcript counts as input. Figure 23 presents the performance differences between the four bin-to-cell assignment methods when combined with the three cell-type annotation methods. Figure 24 shows the normalized confusion matrices to highlight the cell types that may be mistaken with each other.

Cell Annotation Method	Granular label	Pathologist label
Sargent, CellAssign	Epithelial, Enterocytes, Goblet cells, Enteroendocrine cells, Crypt cells	epithelial cells
	B cells, T cells, NK cells	immune cells
	Endothelial, Fibroblast, Smooth muscle cell	stromal cells
CellTypist	CMS1, Mature Enterocytes type 2, Mature Enterocytes type 1, CMS3, CMS4, Stem-like/TA, CMS2, Goblet cells	epithelial cells
	cDC, Regulatory T cells, Neutrophils, Gamma delta T cells, Macrophages, Eosinophils, CD8+ T cells, CD4+ T cells, IgG+ Plasma, Pro-inflammatory, T helper 17 cells, CD19+CD20+ B, Mast cells, NK cells, T follicular helper cells, IgA+ Plasma	immune cells
	Proliferative ECs, Lymphatic ECs, Stromal 2, Stromal 3, Stalk-like ECs, Tip-like ECs, Smooth muscle cells, Stromal 1, Myofibroblasts, Pericytes, Enteric glial cells	stromal cells

Table 5. Look up table mapping the granular cell types to the broader pathologist-provided cell type label.

Cell Annotation Method	Bin-to-Cell Method	Accuracy	Precision	Recall	F-Score
CellAssign	Naive	0.581	0.570	0.581	0.467
	Weight-by-Area	0.581	0.569	0.581	0.468
	Weight-by-Transcript	0.582	0.611	0.582	0.471
	Weight-by-Cluster	0.583	0.570	0.583	0.472
CellTypist	Naive	0.579	0.690	0.579	0.611
	Weight-by-Area	0.581	0.691	0.581	0.612
	Weight-by-Transcript	0.580	0.690	0.580	0.611
	Weight-by-Cluster	0.582	0.692	0.582	0.614
Sargent	Naive	0.684	0.836	0.684	0.740
	Weight-by-Area	**0.708**	0.840	**0.708**	**0.758**
	Weight-by-Transcript	0.688	0.838	0.688	0.744
	Weight-by-Cluster	0.703	**0.841**	0.703	0.754

Table 6. Performance comparison of pipeline with different Bin-to-Cell methods and cell type inference algorithms using the Visium HD Dataset.

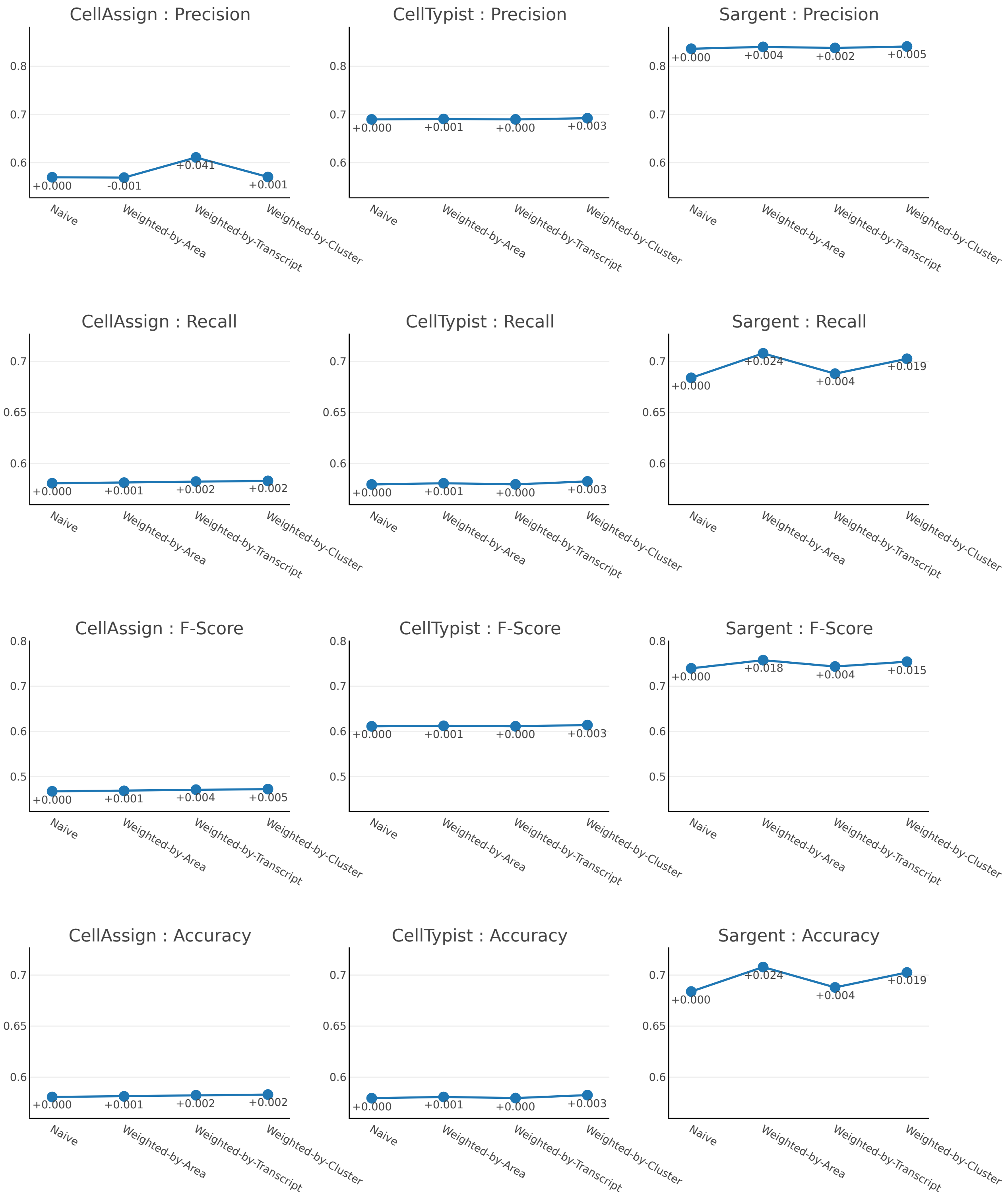

Fig. 23: Precision, recall, F score, and accuracy for the twelve experiments run combining the three cell annotation methods: CellAssign, CellTypist, and Sargent, and the four bin-to-cell assignment methods: Naive, Weighted-by-Area (weighted_by_area), Weighted-by-Transcript (weighted_by_gene), and Weighted-by-Cluster (weighted_by_cluster)

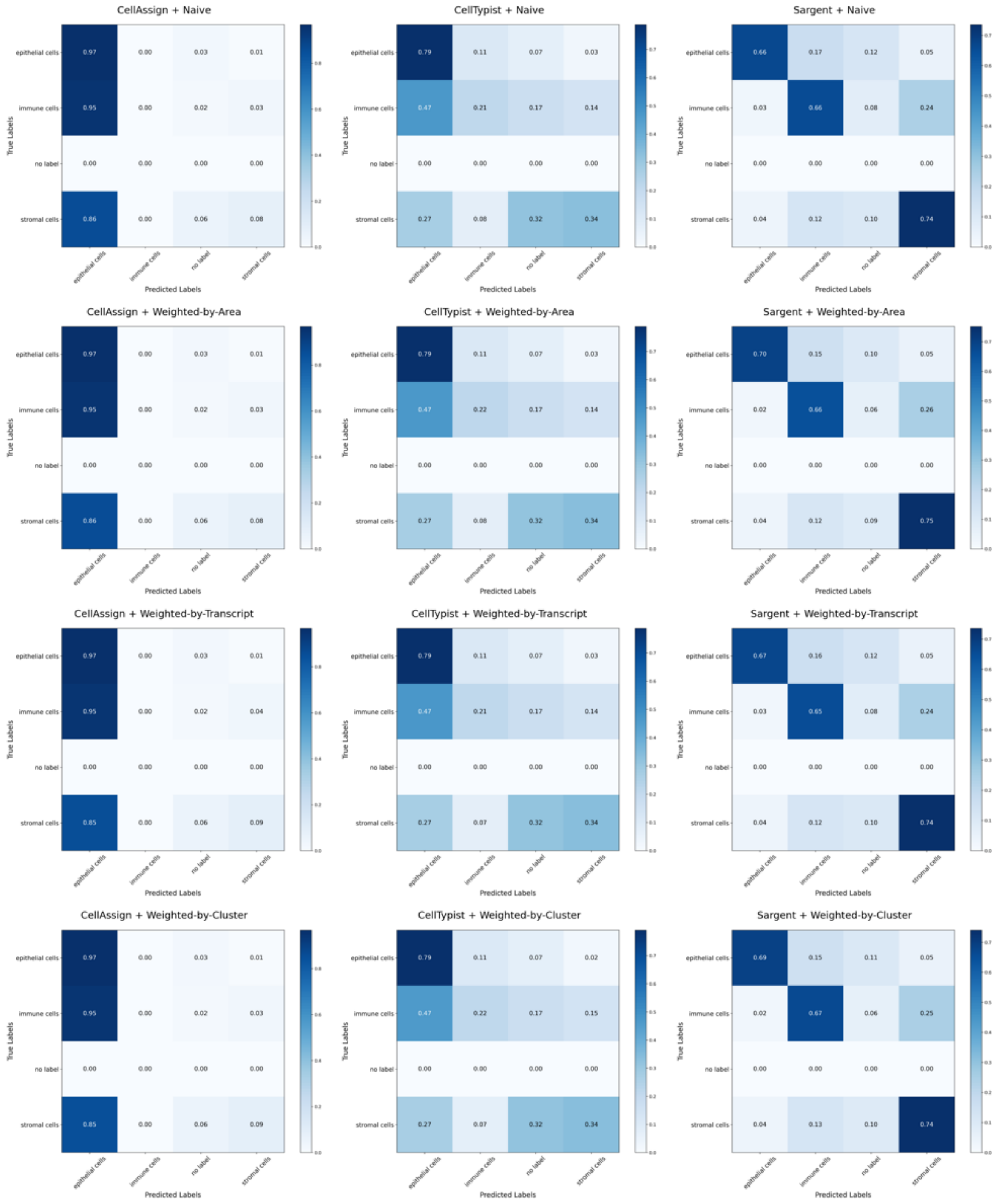

Fig. 24: Confusion matrices for the twelve experiments run combining the three cell annotation methods and four bin-to-cell assignment methods: Naive, Weighted-by-Area, Weighted-by-Transcript, and Weighted-by-Cluster. Showing normalized confusion matrices. Proportions are normalized by the total number of annotated cells per cell type, allowing for an unbiased comparison of prediction performance across cell types, despite the large differences in cell type abundances (12,072 epithelial, 2,748 immune, and 6,171 stromal cells). The color scale reflects the magnitude of these proportions.

3.5. Evaluating Cell-Types in Anatomical Landmarks

This section describes a higher-level analysis of the performance of ENACT compared to the analysis provided in subsection 3.4. Here, the cell-type annotations predicted by ENACT are analyzed in each of the pathologist-annotated anatomical landmarks defined in Table Table 1. Only the cells that are located within each anatomical landmark are considered. Table 7, Table 8, and Table 9 define the gene markers used for this analysis. Results and configurations are available at `https://zenodo.org/records/14748859`.

3.5.1. Human Colorectal Cancer - FFPE

Figures 25-26 show the spatial organization of the predicted cell types and their distributions within the anatomical landmarks for the human colorectal samples. TissUUmaps histopathology web-viewer (Pielawski et al. 2023) is used for visualizing the cell types over the high resolution image.

configurations:

- **Bin-to-cell assignment method:** Weighted-by-Area
- **Cell annotation method:** Sargent
- **Cell or nucleus boundary:** Nuclei boundaries
- **Normalized or raw counts:** Raw counts

Epithelial	Enterocytes	Goblet cells	Entero-endocrine cells	Crypt cells	Endothelial	Fibroblast	Smooth muscle cells	B cells	T cells	NK cells
CDH1	CD55	MANF	NUCB2	HOPX	PECAM1	COL1A1	BGN	CD74	JUNB	S100A4
EPCAM	ELF3	KRT7	FABP5	SLC12A2	CD34	COL3A1	MYL9	HMGA1	S100A4	IL32
CLDN1	PLIN2	AQP3	CPE	MSI1	KDR	COL5A2	MYLK	CD52	CD52	CXCR4
CD2	GSTM3	AGR2	ALCAM	SMOC2	CDH5	PDGFRA	FHL2	PTPRC	PFN1P1	FHL2
	KLF5	BACE2	GCG	OLFM4	PROM1	ACTA2	ITGA1	HLA-DRA	CD81	IL2RG
	CBR1	TFF3	SST	ASCL2	PDPN	TCF21	ACTA2	CD24	EEF1B2P3	CD69
	APOA1	PHGR1	CHGB	PROM1	TEK	FN	EHD2	CXCR4	CXCR4	CD7
	CA1	MUC4	IAPP	BMI1	FLT1		OGN	SPCS3	CREM	NKG7
	PDHA1	MUC13	CHGA	EPHB2	VCAM1		SNCG	LTB	IL32	CD2
	EHF	GUCA2A	ENPP2	LRIG1	PTPRC		FABP4	IGKC	TGIF1	HOPX
					VWF					
					ENG					
					MCAM					
					ICAM1					
					FLT4					

Table 7. Gene markers used to obtain cell type labels using Sargent and CellAssign for Human Colorectal Cancer sample.

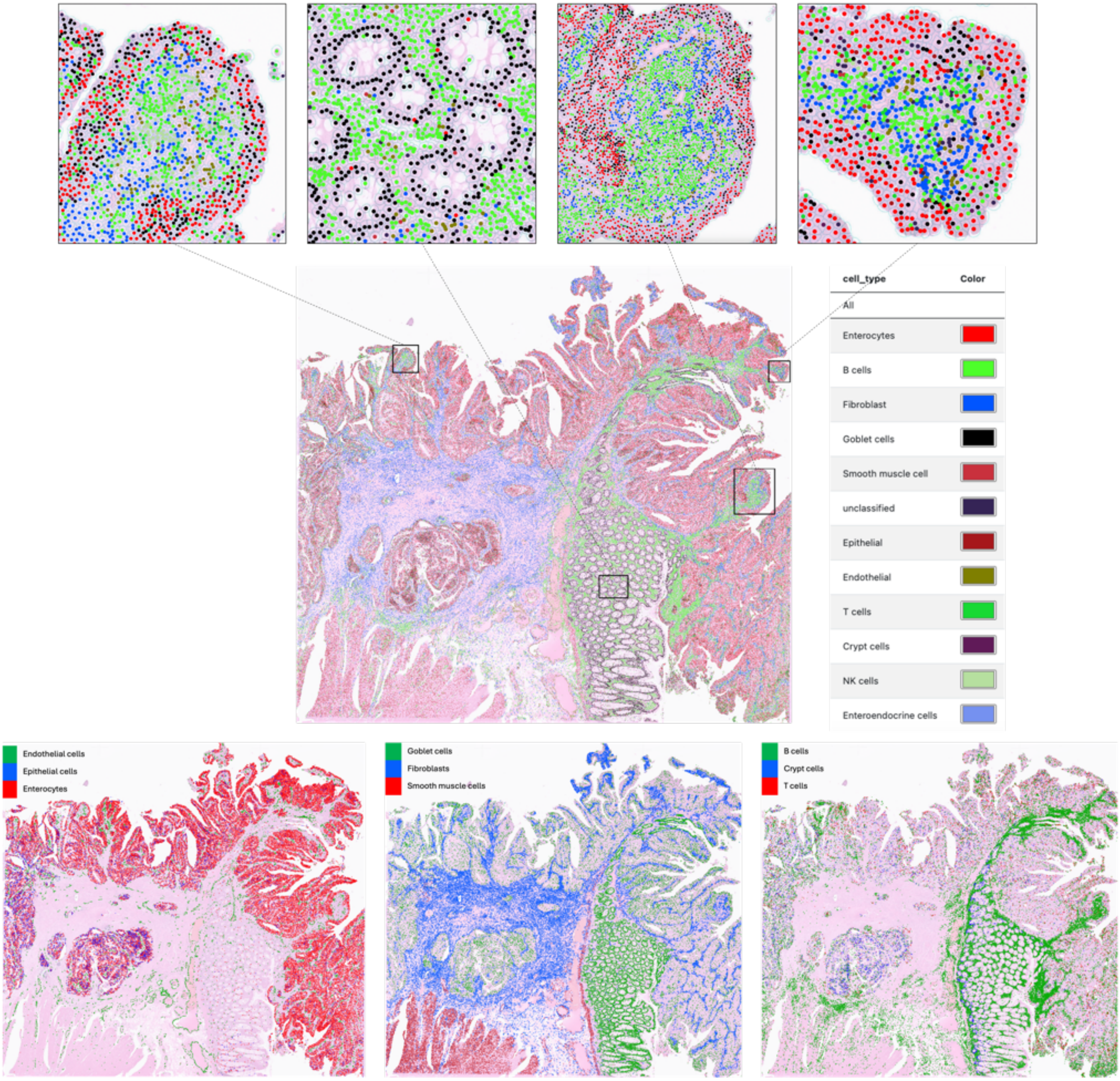

Fig. 25: Spatial distribution of cell types present in the Human Colorectal sample. Each dot represents the centroid of the cell. Predicted cell outlines are shown in blue.

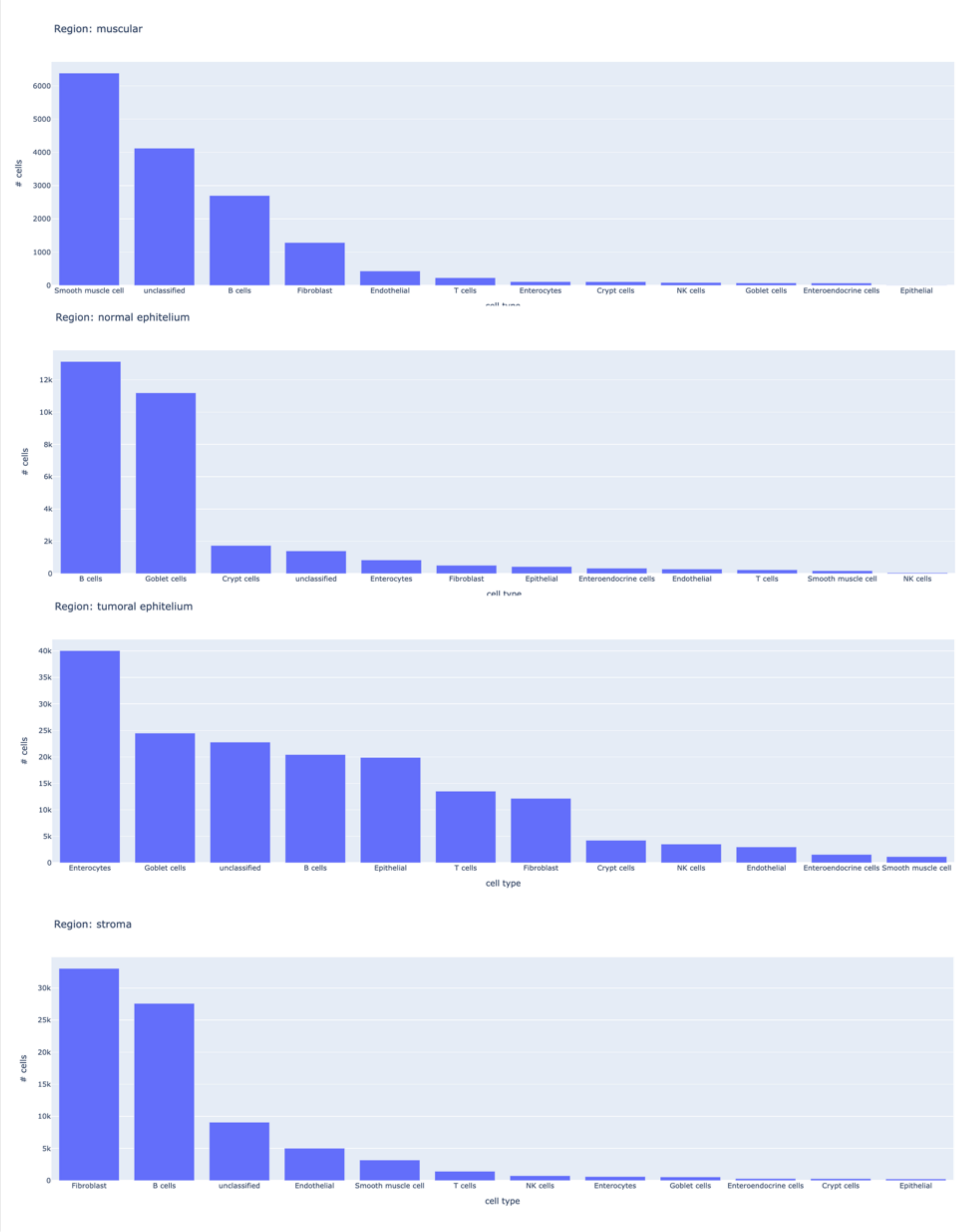

Fig. 26: Distribution of the cell types within the four anatomical landmarks annotated in the Human Colorectal sample.

3.5.2. Mouse Small Intestine - FFPE

Figures 27-28 show the spatial organization of the predicted cell types and their distributions within the anatomical landmarks for the mouse small intestine sample.
ENACT configurations:

- **Bin-to-cell assignment method:** Weighted-by-Area
- **Cell annotation method:** Sargent
- **Cell or nucleus boundary:** Nuclei boundaries
- **Normalized or raw counts:** Raw counts

Enterocytes	Goblet cells	Entero-endocrine cells	Paneth cells	Crypt cells	Smooth muscle cells	B cells	T cells	NK cells
Cbr1	Manf	Fabp5	Gpx2	Prom1	Bgn	Cd52	Cd81	Ctla2A
Plin2	Krt7	Cpe	Fabp4	Hopx	Myl9	Bcl11A	Junb	Ccl4
Gls	Ccl9	Enpp2	Lyz1	Msi1	Pcp4L1	Ebf1	Cd52	Cd3G
Plin3	Muc13	Chgb	Kcnn4	Olfm4	Itga1	Cd74	Ptprcap	Ccl3
Dab1	Phgr1	Alcam	Lgals2	Kcne3	Nrp2	Ptprc	H2-Q7	Nkg7
Pmepa1	Cdx2	Chga	Guca2B	Bmi1	Mylk	Pold4	Ccl6	Lat
Acsl5	Aqp3	Pax6	Lgr4	Axin2	Ehd2	Ighm	Bcl2	Dusp2
Hmox1	Creb3L1	Neurod1	Defa24	Kcnq1	Fabp4	Cd14	Maff	Itgam
Abcg2	Guca2A	Cck	Il4Ra	Ascl2	Acta2	Creld2	Ccl4	Fhl2
Cd36	Klk1	Isl1	Guca2A	Lrig1	Ogn	Fli1	Ccl3	Ccl5

Table 8. Gene markers used to obtain cell type labels using Sargent and CellAssign for Mouse Small Intestine sample.

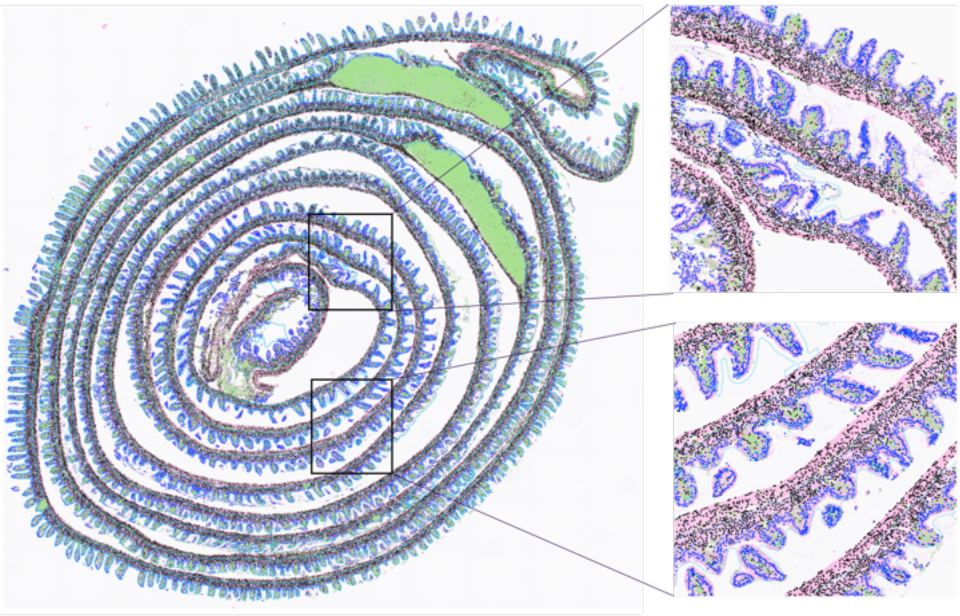

cell_type	Counts	Color
All	392234	
B cells	94448	
Crypt cells	3091	
Enterocytes	15409	
Enteroendocrine cells	950	
Goblet cells	83846	
NK cells	1978	
Paneth cells	80831	
Smooth muscle cells	67796	
T cells	1546	
unclassified	42339	

Fig. 27: Spatial distribution of cell types present in the Mouse Small Intestine sample. Each dot represents the centroid of the cell.

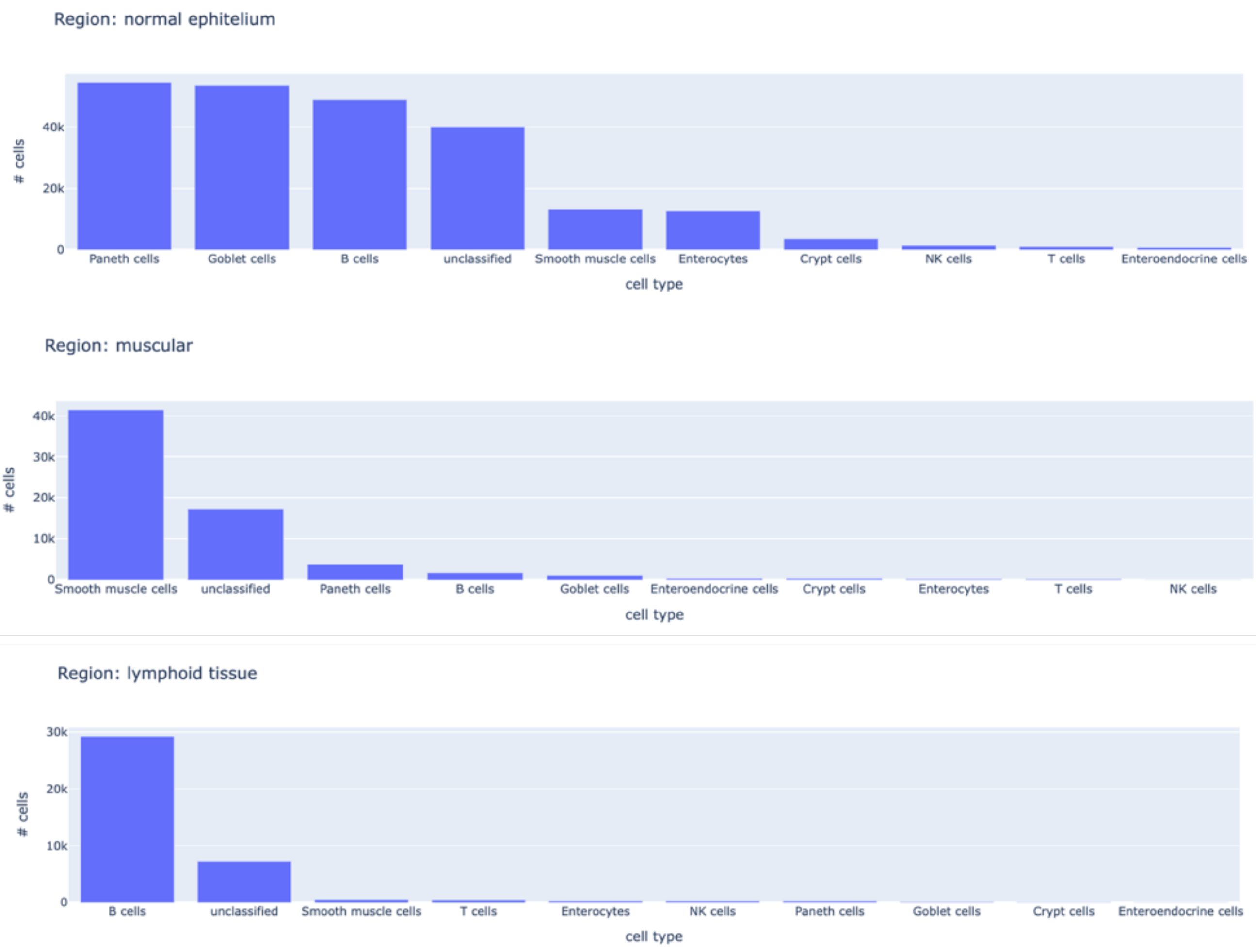

Fig. 28: Distribution of the cell types within the four anatomical landmarks annotated in the Mouse Small Intestine sample.

3.5.3. Human Lung Cancer - FFPE

ENACT configurations:

- **Bin-to-cell assignment method:** Weighted-by-Area
- **Cell annotation method:** Sargent
- **Cell or nucleus boundary:** Cell boundaries
- **Normalized or raw counts:** Raw counts

Gene markers used to predict cell types with Sargent are presented in Table 9. These markers were directly adopted from Sargent's supplementary material for lung samples (Nouri et al. 2023).

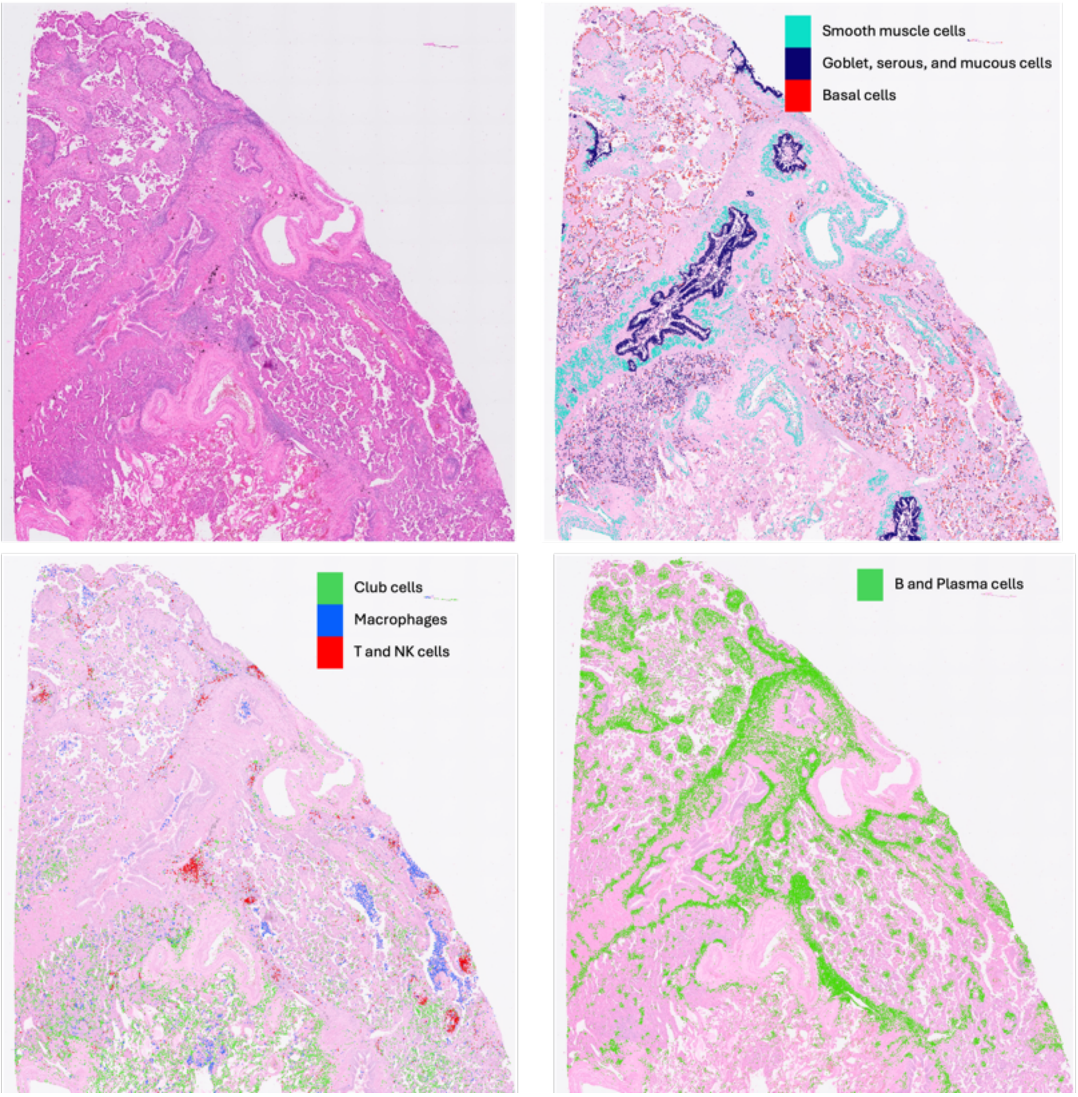

Fig. 29: Spatial distribution of various cells detected in a human lung cancer sample (FFPE) with ENACT using Weight-by-Area for bin to cell assignment and Sargent for cell annotation.

Macrophages	B and Plasma Cells	T Cells and NK Cells	Neutrophils	Basophils	Endothelial Cells	Adventitial Cells	Fibroblasts	Mesothelial Cells
CD4	CD79A	CD4	S100A8	CD63	CD34	CD34	COL3A1	MSLN
MS4A7	CD79B	CD3D	S100A12	CD203C	EGFL7	ANPEP	COL5A2	UPK3B
VCAN	MS4A1	CD3E	FCGR3B	CD123	EMCN	UAP1	FN1	WT1
FCN1	JCHAIN	CD3G	LILRA5	CLC	FLT1	VIT	LUM	CALB2
FCGR3A	TNFRSF17	IL7R	G0S2	MS4A3	KDR	MFAP5	LRP1	VCAM1
ITGAM	DERL3	CD8A		TCN1	TEK	PCOLCE2	PDGFRA	MEDAG
CD1C	FCRL5	CD8B2		CPA3	VWF	GFPT2	TCF21	GAS1
ITGAX	MZB1	FOXP3		HDC	ACKR1	DPT	SCARA5	HAS1
LILRB2	VPREB3	TIGIT		GATA2	CA4	PDGFRA		C1S
CD14	IGHA1	CD27		MS4A2		LUM		C2
	IGHM	NCAM1		IL4		SCARA5		CFB
	IGHG1	KLRF1		GCSAML				
	CD27	GNLY		TPSAB1				
**Mesothelial Cells**	**Plasma Cells**	**Smooth Muscle Cells**	**Lung Ciliated Cells**	**Goblet Serous and Mucous Cells**	**Pulmonary Ionocytes**	**Basal Cells**	**Club Cells**	**Alveolar Type 1 and Type 2 Cells**
MSLN	KCNK3	PLN	FOXJ1	MUC5B	CFTR	AQP3	SCGB3A2	AGER
UPK3B	CDH6	DES	TUBB1	MUC5AC	FOXI1	KRT5	CYP2B7P	CAV1
WT1	COX4I2	TNNT2	TP73	SPDEF	ASCL3	KRT14	MGP	CLDN18
CALB2	COX4I2	ACTG2	CCDC78	SCGB1A1	ATP6V1G3	KRT15	SFTPC	CAV2
VCAM1	PDGFRB	ATP1A2	CAPS	SCGB1A1	HEPACAM2	TP63	SFTPD	ALOX15B
MEDAG	CSPG4	MYH11		BPIFB1		DAPL1		LRRK2
GAS1	TRPC6	TAGLN		LTF		MIR205HG		ROS1
HAS1	RGS5	ACTA2		ANPEP		EYA2		SFTPA1
C1S		CALD1		EPCAM		CYP24A1		CSF3R
C2		NOTCH3		IL10		KRT17		
CFB				LTF				

Table 9. Gene markers used to obtain cell type labels using Sargent for Human Lung Cancer sample.

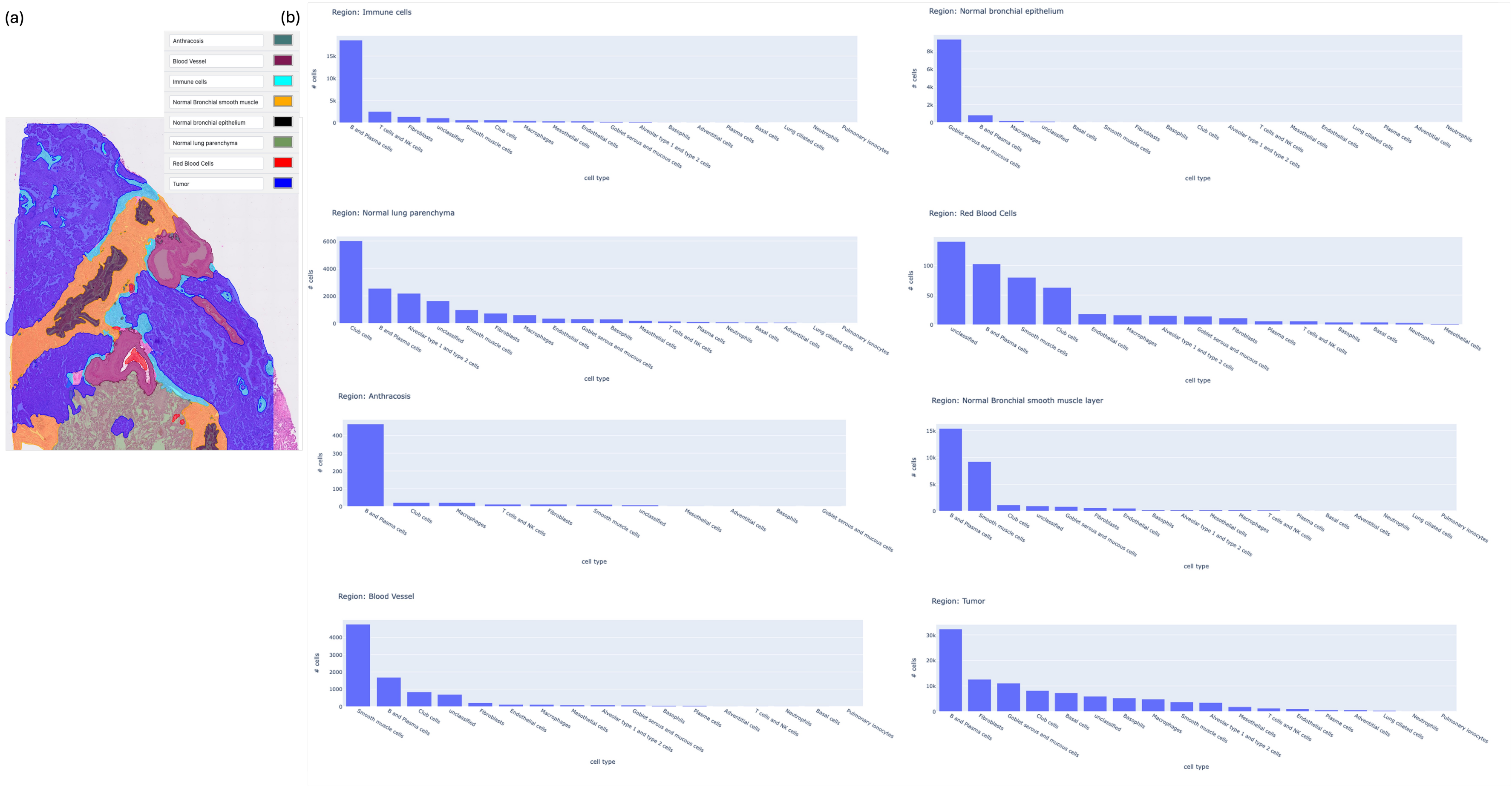

Fig. 30. (a) Expert-labeled anatomical landmarks for human lung cancer sample. (b) Cell types predicted within each anatomical landmark.

3.5.4. Human Breast Cancer - Fresh Frozen

ENACT configurations:

- **Bin-to-cell assignment method:** Weighted-by-Area
- **Cell annotation method:** CellTypist
- **Cell or nucleus boundary:** Cell boundaries
- **Normalized or raw counts:** Raw counts

CellTypist is employed to predict cell types using its pre-trained model for the human breast, which was trained on the Human Breast Cell Atlas (HBCA): [9].

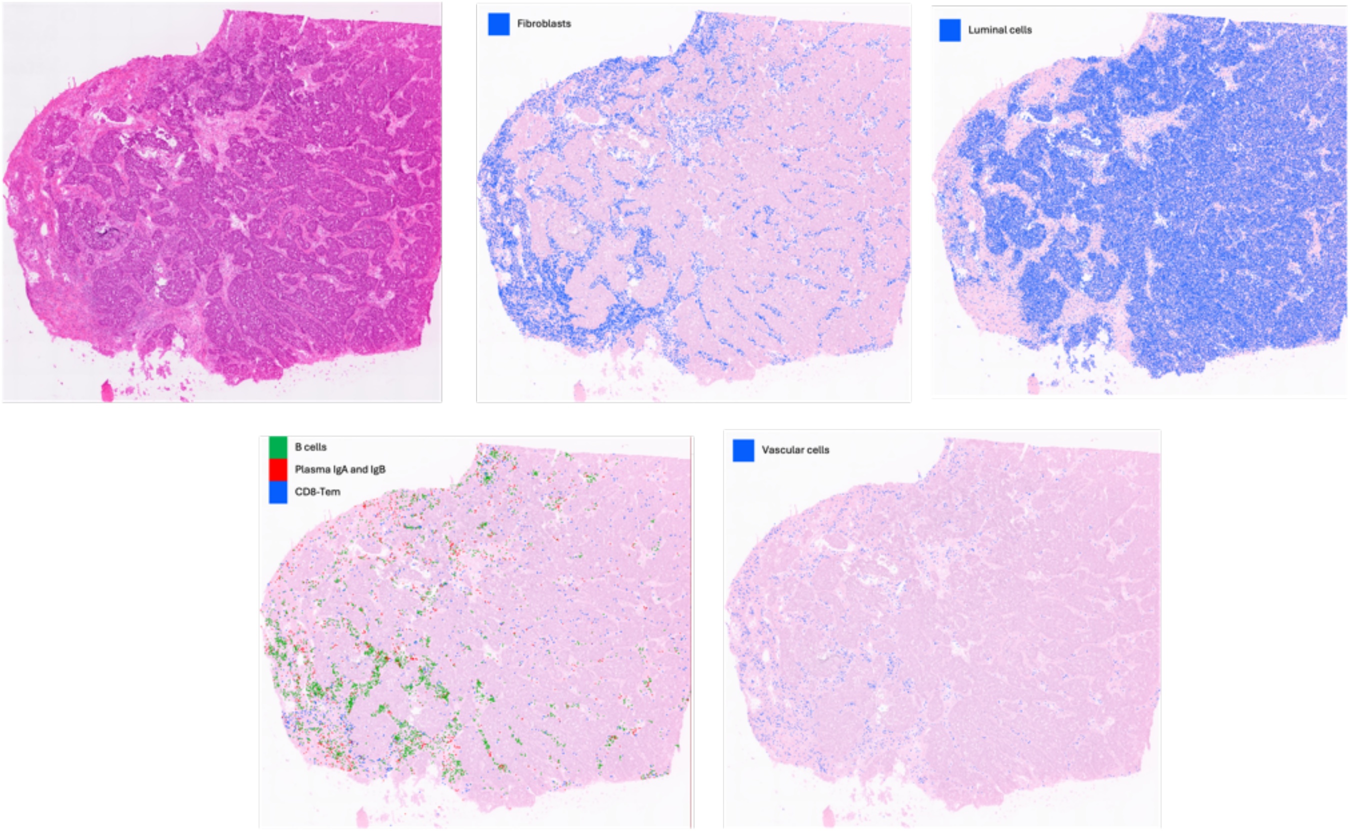

Fig. 31: Spatial distribution of various cells detected in a human breast cancer (fresh frozen) sample with ENACT using Weight-by-Area for bin to cell assignment and CellTypist for cell annotation.

[9] https://doi.org/10.1038/s41586-023-06252-9

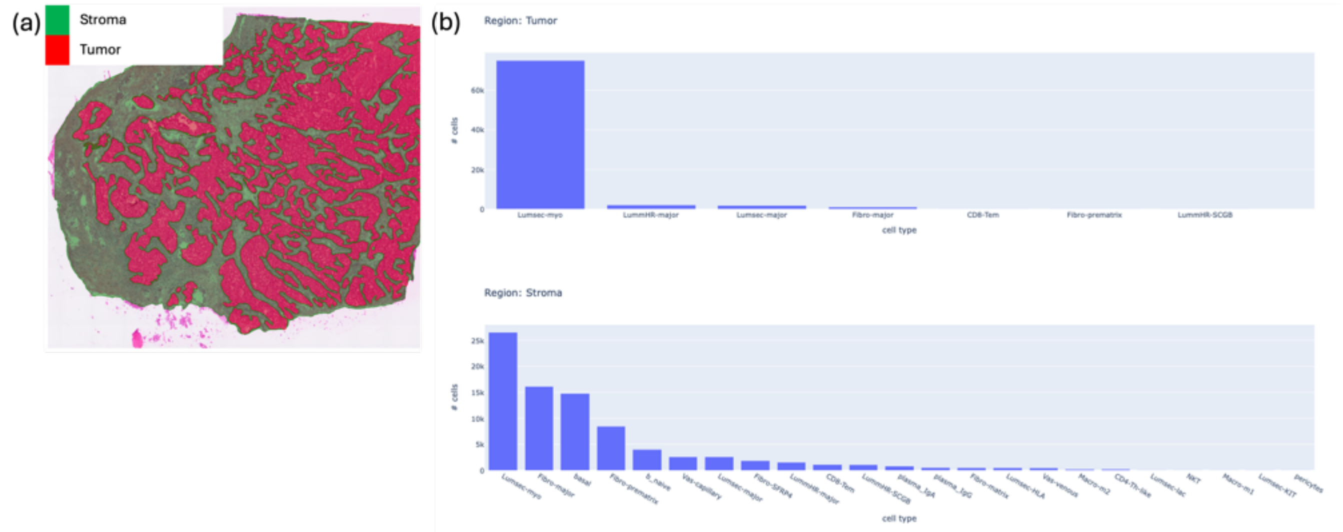

Fig. 32: (a) Expert-labeled anatomical landmarks for human breast cancer sample. (b) Cell types predicted within each anatomical landmark.

3.5.5. Human Tonsil - Fresh Frozen

ENACT configurations:

- **Bin-to-cell assignment method:** Weighted-by-Area
- **Cell annotation method:** CellTypist
- **Cell or nucleus boundary:** Cell boundaries
- **Normalized or raw counts:** Raw counts

CellTypist is employed to predict cell types using its pre-trained model for the human tonsil, which was trained on the Human Tonsil Atlas:[10].

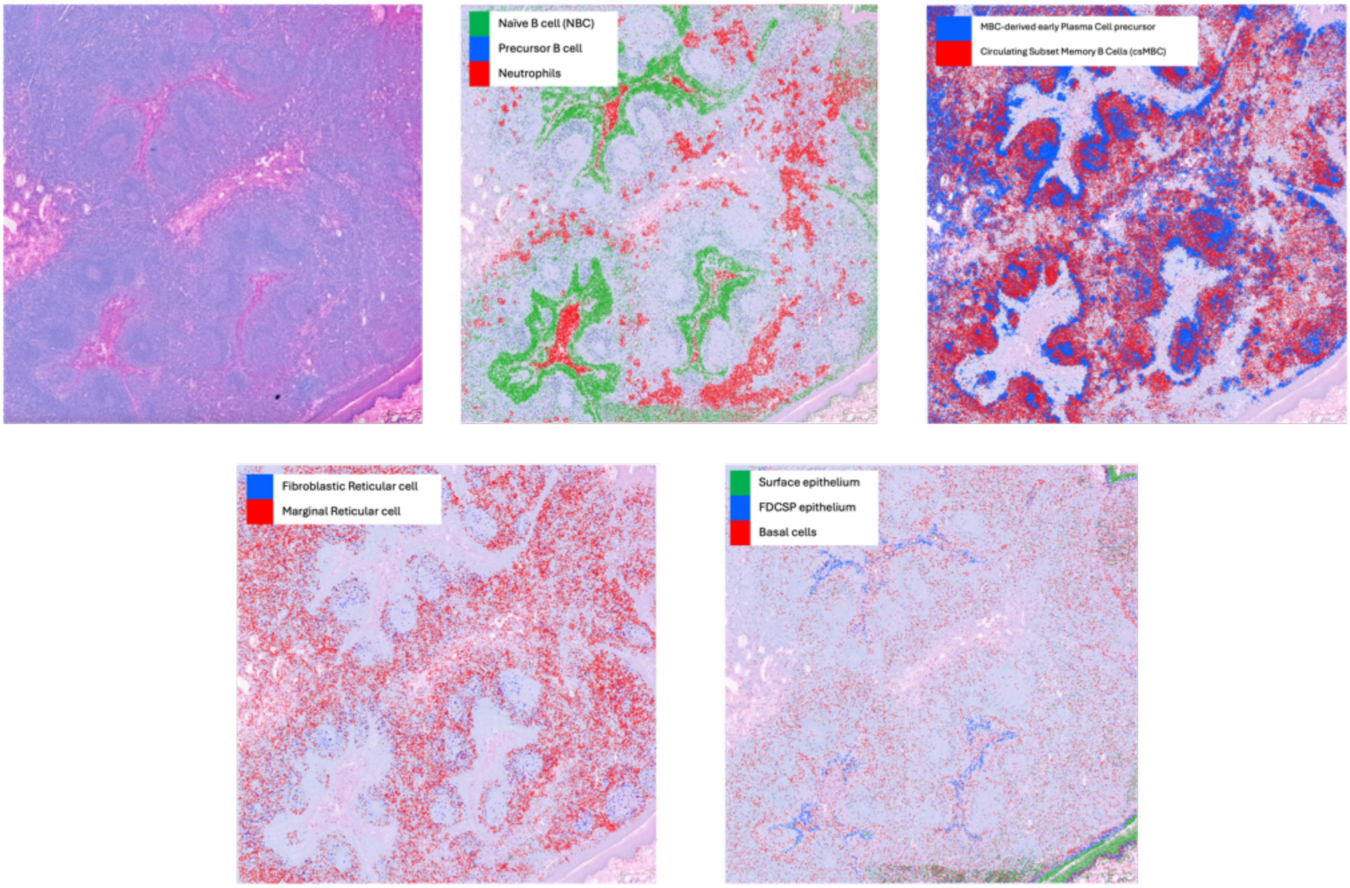

Fig. 33: Spatial distribution of various cells detected in a human tonsil (fresh frozen) sample with ENACT using Weight-by-Area for bin to cell assignment and CellTypist for cell annotation.

[10] https://doi.org/10.1016/j.immuni.2024.01.006

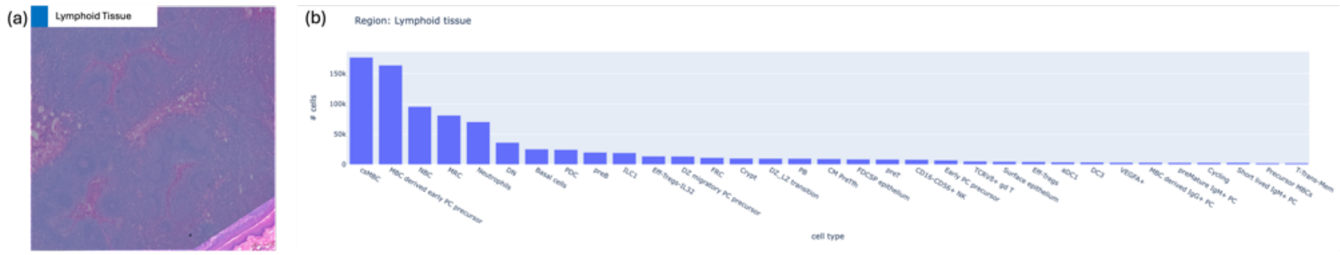

Fig. 34: (a) Expert-labeled anatomical landmarks for human tonsil sample. (b) Cell types predicted within each anatomical landmark.

4. Comparison of ENACT and Bin2cell

ENACT shares some similarities with Bin2cell (Polanski et al. 2024) as both packages allow analyzing Visium HD data. Bin2Cell allows to identify cells in Visium HD data and capture its transcripts information by employing an all-or-nothing approach, assigning each bin exclusively to a single cell. To identify cells, it combines both imaging and transcriptomics data and relies on CellTypist. On the other hand, ENACT ia a unified pipeline which includes cell segmentation, bin-to-cell assignment, and cell-type annotation, and TissuUUmaps visualization all in one package. Conversely to Bin2Cell, it offers a more flexible approach to transcript assignment and cell type assignment. Table 10 shows that Bin2Cell and ENACT present similar performance when CellTypist is used as ENACT's cell assigning engine and how ENACT+Sargent outperform Bin2Cell. Results show how normalization can condition cell assignment outcomes and that it needs to be set up in alignment to the selected cell classification algorithm.

4.1. Dataset

Evaluation is performed on a subset of 15,554 manually labeled pathologist annotations from four patches of a human colorectal cancer sample as described in Table 2. While the original dataset contained approximately 20,991 cells, the default segmentation parameters of Bin2cell missed some cells. To ensure a fair comparison, the evaluation was restricted to the 15,554 cells that were successfully segmented by both Bin2cell and ENACT's.

4.2. Comparing ENACT and Bin2cell on Cell Type Prediction

4.2.1. Impact of Normalization

The authors of Bin2cell highlight an artifact observed in Visium HD data where the 2 μm bins exhibit a capture disparity along rows and columns, resulting in a characteristic 'striped' effect when visualizing total transcript counts per bin across tissue sections. To address this, they propose a row-column normalization procedure, referred to as 'destripe' normalization. In their method, each bin's total count is divided by a quantile (default: 0.99) for its corresponding row, with the process repeated for columns. The resulting normalization factors are then scaled back into count space by multiplying them with the specified global per-bin quantile. This procedure is reported to reduce the 'striped' effect.

The bin-to-cell assignment methods proposed in ENACT rely on raw transcript counts and the proportions of these counts shared among cells or clusters within a bin. As such, these assignment steps are not directly influenced by linear normalization techniques. However, normalization is critical for subsequent downstream analyses, where different tools handle it in distinct ways. For example, CellTypist includes its own internal normalization procedure, CellAssign integrates normalization into its probabilistic modeling framework, and Sargent is agnostic to normalization. In this context, ENACT provides the appropriate normalization approach required by each method, as specified in their respective documentation or publications.

To assess the impact of 'destripe' normalization on cell type prediction, an ablation study is performed to compare ENACT's performance with and without 'destripe' normalization. The results are presented in Table 10.

Using CellTypist for cell annotation, 'destripe' normalization improved the F1-score from 0.612 to 0.618 and the accuracy from 0.587 to 0.592. However, when using Sargent, 'destripe' normalization slightly reduced performance. ENACT includes the option to apply 'destripe' normalization, allowing users to enable it based on their specific analysis needs.

4.2.2. Impact of using cell boundaries versus nuclei boundaries

Bin2cell segments nuclei in a two-stage process combining image and gene information. The nuclei are discretized into bins and expanded by 2 μm to approximate cell boundaries. ENACT allows users to define the distance for nuclei expansion to obtain cell boundaries. ENACT starts by segmenting nuclei using Stardist, followed by generating Voronoi polygons based on nuclei centroids. Nuclei boundaries are then expanded within their respective Voronoi polygons using the user-defined distance to ensure non-overlapping cell boundaries as shown in Figure 35.

Nuclei expansion significantly improves ENACT's performance. With Sargent for cell-type annotation, nuclei expansion results in a 2.6% improvement in F1-score and a 3.8% improvement in accuracy. Using CellTypist, the improvements are more modest, with a 0.60% gain in F1-score and a 0.50% drop in accuracy.

When comparing ENACT (with nuclei expansion and Sargent) to the default Bin2cell implemenation (nuclei expansion with CellTypist), ENACT achieves an F1-score of 0.787 and accuracy of 0.761, outperforming Bin2cell's 0.617 F1-score and 0.591 accuracy.

Bin-to-Cell Method	Boundary Type	Normalization	Accuracy	Precision	Recall	F-Score
Bin2cell	Cell boundary	‘destripe’ normalization	0.591	0.680	0.591	0.617
ENACT (CellTypist)	Nucleus boundary	None	0.588	0.667	0.588	0.608
	Cell boundary	None	0.587	0.676	0.587	0.612
	Cell boundary	‘destripe’ normalization	**0.592**	**0.682**	**0.592**	**0.618**
ENACT (Sargent)	Nucleus boundary	None	0.723	0.830	0.724	0.761
	Cell boundary	None	**0.761**	**0.831**	**0.761**	**0.787**
	Cell boundary	‘destripe’ normalization	0.756	0.828	0.756	0.782

Table 10. Performance comparison of ENACT and Bin2cell on cell type prediction using the pathologist annotations of the Human Colorectal Cancer sample. Here ‘cell boundary’ refers to the boundaries obtained after expanding the nuclei boundaries by 2μm all around.

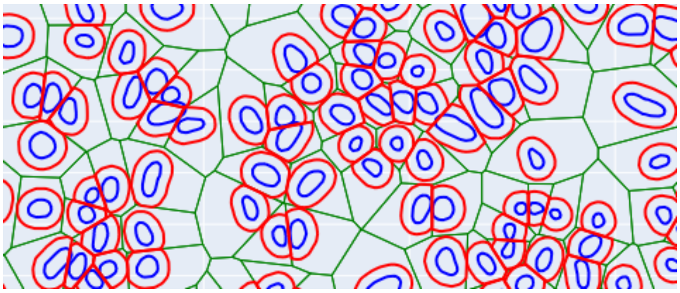

Fig. 35: Nuclei expansion to obtain cell boundaries. The original nuclei boundaries (blue) from Stardist are used to create a Voronoi diagram (green). Each nucleus is expanded within its Voronoi polygon by the user-defined distance to yield non-overlapping cell boundaries (red).

This represents an absolute improvement of 0.17 in F1-score and accuracy, a relative improvement of 27.6% in terms of F1-score, and a relative improvement of 28.8% in accuracy.Table 10 shows a full comparison of ENACT's performance with and without nuclei expansion.

5. ENACT's Considerations and Limitations

5.1. Impact of Transcript Count Sparsity

Both CellAssign, CellTypist and Sargent were designed primarily for single-cell data, where transcriptomic signals are generally stronger and less sparse than in spatial transcriptomics, such as with Visium HD data. Methods like CellAssign or CellTypist may be more sensitive to the sparsity and lower signal in spatial data; the observed performance in this context should not undermine its effectiveness when applied to single-cell datasets.

5.2. Gene Marker Selection

ENACT relies on cell type annotation algorithms that use a list of gene markers to identify the different cell types present in the data. Several factors should be considered when defining the list of markers to be used:

5.2.1. Cell Type Granularity

Sargent paper notes that excessive granularity in cell type-specific gene sets can complicate annotation. A common approach is to perform initial annotation at a broader level and refine it within subgroups for greater granularity. CellAssign performs effectively with highly granular annotations, even when cell types are over-specified (see CellAssign Supplementary Note, Figure 2D) [11].

In CellTypist, the granularity of cell types is determined by the properties of the pre-trained models used in the analysis. A potential limitation arises when the desired cell type is not included in the list of cell types that the CellTypist model is trained to predict. In such scenario, users are advised to train new CellTypist models or to rely on the other two cell assignment methods.

5.2.2. Suboptimal Gene Markers

Poor classification performance can arise from the use of suboptimal gene markers, especially when markers do not sufficiently distinguish the cell types of interest. Additionally, the sparsity of transcript data in spatial transcriptomics can exacerbate misclassification due to low signal-to-noise ratios, hindering accurate cell type assignment.

5.2.3. Number of Gene Markers

Sargent's supplementary documentation suggests that 3-27 markers were used per cell type (see Sargent Supplementary Material) [12]. Notably, the Sargent method is not overly sensitive to the over-specification of marker gene sets. For CellAssign, the authors used 30-68 marker genes across a total of 84 genes to identify human embryonic stem cells (hESCs). They also demonstrated that CellAssign can perform well with as few as 3 specific marker genes, even at modest expression levels (see CellAssign Supplementary Note 2.4).

Therefore, a minimum number of 3 gene markers is recommended for CellAssign and Sargent. We refer the readers to CellAssign and Sargent's documentation for further details about marker gene selection.

5.3. Impact of Tissue Density

The effectiveness of bin-to-cell methods is influenced by tissue density. In regions with low cell density or cell sparsity, differences between bin-to-cell methods are minimal. For instance, in the synthetic Xenium-based dataset, using nuclei boundaries with greater spacing between cells shows little impact from the choice of method. However, in dense tissues, where a larger proportion of 2μm bins overlap multiple nuclei, weighted methods demonstrate a clear advantage. A similar trend is observed in the SeqFISH+ dataset, where sparse cell distributions lead to similar performance across all methods, as most bins are uniquely assigned to individual cells.

Expanding nuclei to include cell body areas increases overlaps in dense regions, further enhancing the benefits of weighted approaches. In sparse tissues, where overlaps are rare, the choice of method has a smaller effect.

5.4. Cell Segmentation Limitations

Cell segmentation is a crucial step in ENACT, as the bin-to-cell assignment depends on accurate identification of nuclei or cell boundaries to calculate cell-wise transcript counts, which are then used for cell type annotation. Under-segmentation can cause cells to be missed during the segmentation process. Conversely, over-segmentation can result in the fragmentation of cells into smaller parts, each with a reduced transcript count, potentially leading to misclassification or over-counting of cell types.

We recommend that users test the cell segmentation step with various Stardist parameter configurations, carefully reviewing the cell outlines on TissUUmaps to ensure optimal segmentation performance.

A potential future direction to minimize the limitations of image-based segmentation could involve incorporating a secondary segmentation step based on transcript counts, similar to Bin2Cell (Polanski et al. 2024) or to integrate segmentation-free methods such as TopACT (Benjamin et al. 2024) Ficture (Si et al. 2024) and Sainsc (Müller-Bötticher et al. 2024) into a hybrid pipeline that combines both image-based and gene-based approaches.

[11] https://static-content.springer.com/esm/art%3A10.1038%2Fs41592-019-0529-1/MediaObjects/41592_2019_529_MOESM1_ESM.pdf

[12] https://ars.els-cdn.com/content/image/1-s2.0-S2215016123001966-mmc1.xlsx

6. ENACT Usage Guide

Please refer to ENACT's GitHub page https://github.com/Sanofi-Public/enact-pipeline for a full guide on how to run ENACT on Visium HD data. The `ENACT_outputs_demo.ipynb` (Figure 36) notebook provides a comprehensive, step-by-step guide on how to access and analyze output data from ENACT. The notebook covers the following topics:

- **Loading the AnnData object in Python**
  Learn how to load the main data structure for single-cell analysis.
- **Extracting cell types and their spatial coordinates**
  Access information about cell types and their positions in the tissue.
- **Determining the number of shared and unique bins per cell**
  Explore metrics that characterize the bin and cell relationships.
- **Accessing and visualizing the number of transcripts per cell**
  Visualize and analyze transcriptional activity across cells.
- **Identifying the top-n expressed genes in the sample**
  Retrieve the most highly expressed genes in your dataset.
- **Generating interactive plots**
  Visualize cell boundaries and cell types within the tissue using interactive visualizations.
- **Performing downstream analysis**
  Run a sample analysis, such as neighborhood enrichment analysis, using external packages like **Squidpy**.

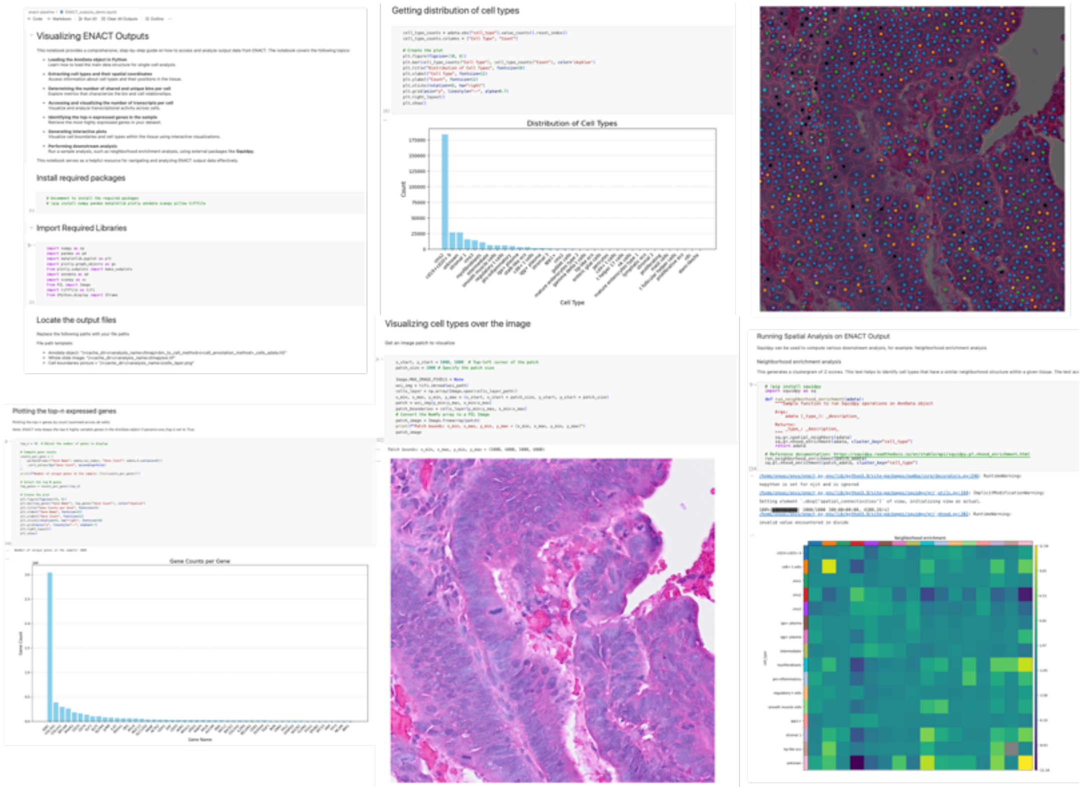

Fig. 36: Screenshots from `ENACT_outputs_demo.ipynb` notebook.

References

Benjamin, Katherine et al. (2024) "Multiscale topology classifies cells in subcellular spatial transcriptomics". In: *Nature*, pp. 1–7.

Eng, Chee Huat (Linus) and Long Cai (May 2019) *NIH3T3 point locations for RNA seqFISH+ experiments*. Zenodo. URL: https://doi.org/10.5281/zenodo.2669683.

Müller-Bötticher, Niklas et al. (2024) "Sainsc: A Computational Tool for Segmentation-Free Analysis of In Situ Capture Data". In: *Small Methods*, p. 2401123.

Nouri, Nima et al. (2023) "A marker gene-based method for identifying the cell-type of origin from single-cell RNA sequencing data". In: *MethodsX* 10, p. 102196.

Pielawski, Nicolas et al. (2023) "TissUUmaps 3: Improvements in interactive visualization, exploration, and quality assessment of large-scale spatial omics data". In: *Heliyon* 9.5. Publisher: Elsevier.

Polanski, Krzysztof et al. (2024) "Bin2cell reconstructs cells from high resolution Visium HD data". In: *Bioinformatics*, pp. 2024–06.

Rizzetto, Simone et al. (2017) "Impact of sequencing depth and read length on single cell RNA sequencing data of T cells". In: *Scientific reports* 7.1, p. 12781.

Si, Yichen et al. (2024) "FICTURE: scalable segmentation-free analysis of submicron-resolution spatial transcriptomics". In: *Nature Methods* 21.10, pp. 1843–1854.
